# Supplementary material for: Inverted signaling by bacterial chemotaxis receptors
Source: Nat Commun. 2018 Jul 26;9:2927. doi: 10.1038/s41467-018-05335-w (PMC6062612; doi:10.1038/s41467-018-05335-w)
Supplement: Supplementary file 1 — Supplementary Information [file 41467_2018_5335_MOESM1_ESM.pdf]

Supplementary Information

Inverted signaling by bacterial chemotaxis receptors

Shuangyu Bi, Fan Jin, and Victor Sourjik

Supplementary Figures

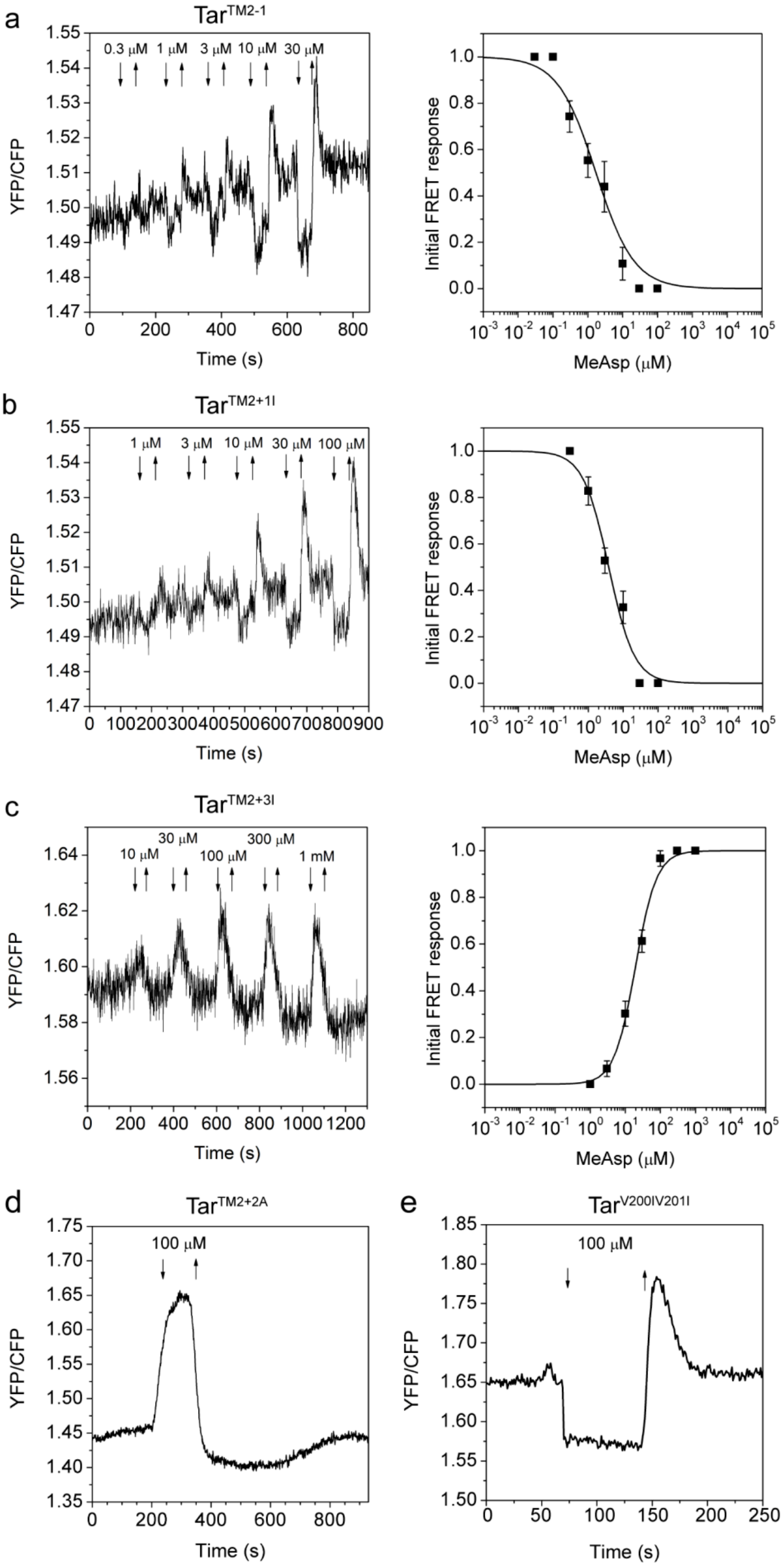

Supplementary Figure 1. FRET responses of the wild-type and mutant Tar expressed in CheR^{+}CheB^{+} strain VS181. (a-c) FRET measurements and dose responses of buffer-adapted *E. coli* cells expressing Tar^{TM2-1} (a), Tar^{TM2+1I} (b), Tar^{TM2+3I} (c), Tar^{TM2+2A} (d) or Tar^{V200IV201I} (e) as a sole receptor as well as the FRET pair to a stepwise addition (down arrow) and subsequent removal (up arrow) of the indicated concentrations of MeAsp. The data were normalized to the saturated response. Error bars indicate standard deviation of three independent replicates using different cell growth cultures. Wherever invisible, error bars are smaller than the symbol size.

a Wild-type Tar

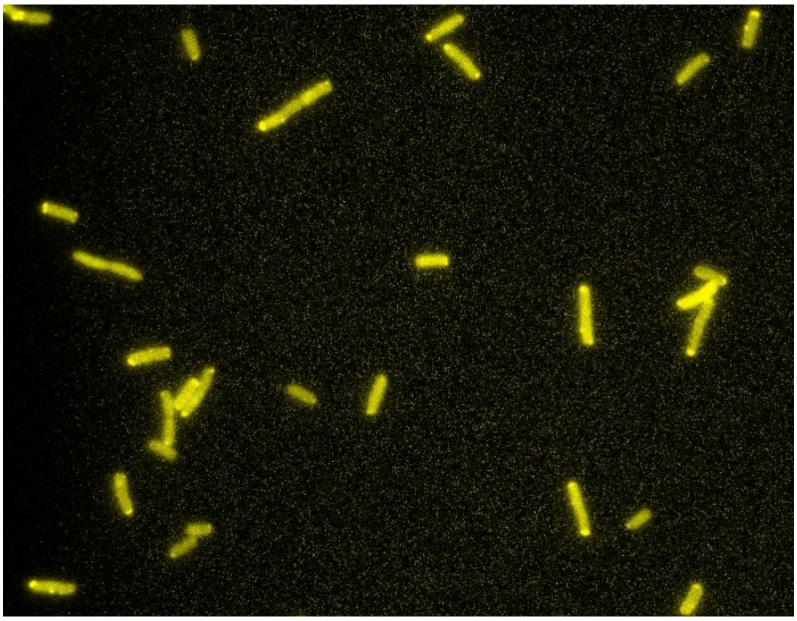

b Tar^{TM2+2I}

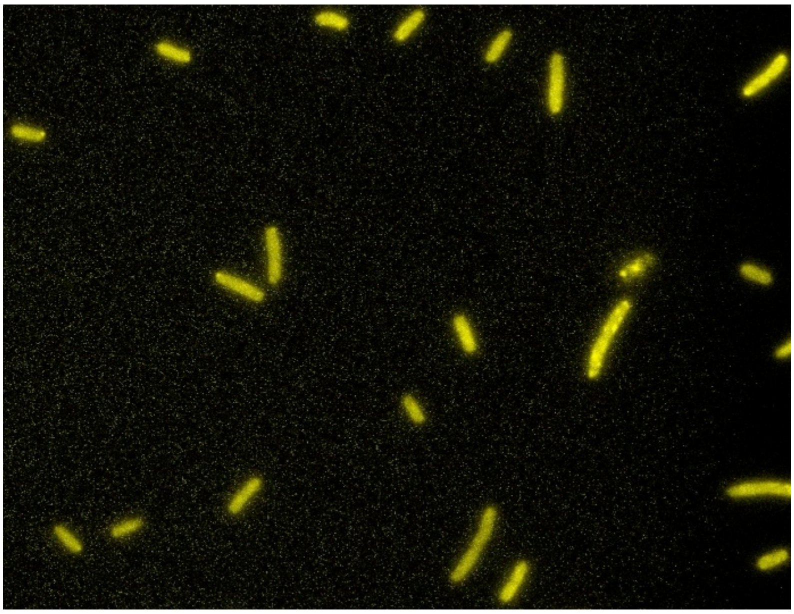

Supplementary Figure 2. Clustering of wild-type and mutant Tar. Wild-type Tar (a) and Tar^{TM2+2I} (b) were co-expressed in receptorless CheR^{+}CheB^{+} strain UU1250 together with catalytically inactive YFP-CheR^{D154A} that marks clusters by binding to receptors.

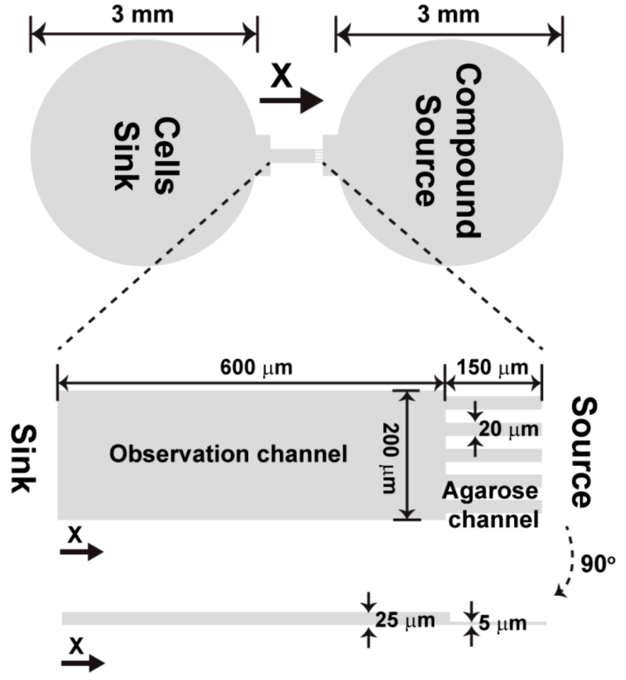

Supplementary Figure 3. Diagram of the microfluidic device. The sink side pore for adding cells, source side pore for adding compound solutions, top and the side view of the observation channel and agarose gel channel are shown.

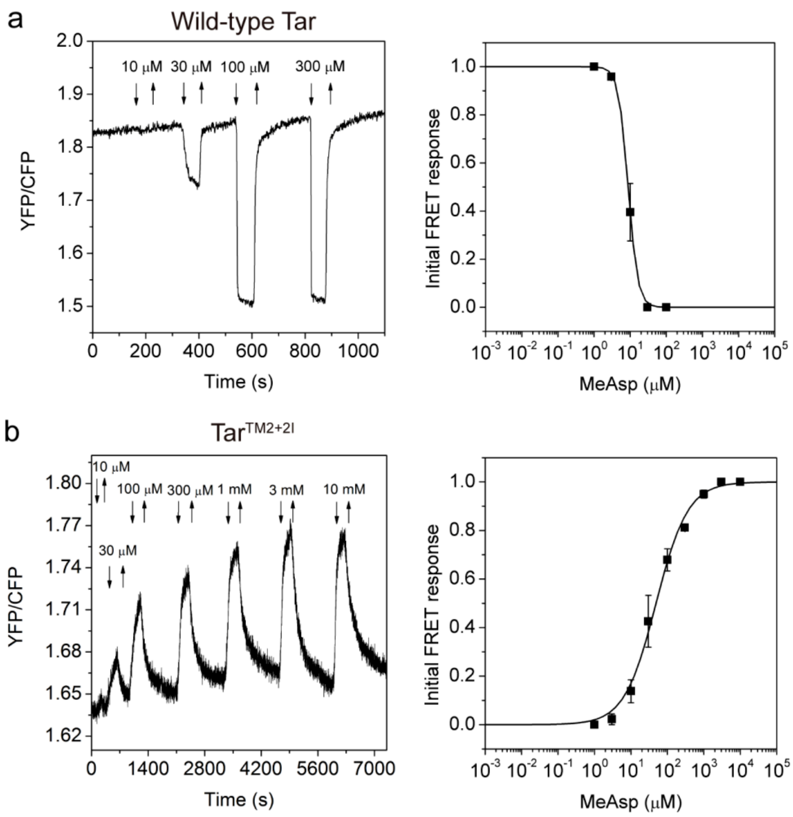

Supplementary Figure 4. FRET responses of the wild-type and mutant Tar expressed in *cheRcheB* strain VH1. (a,b) FRET measurements and dose responses of buffer-adapted *E. coli* cells expressing the wild-type Tar (a) or Tar^{TM2+2I} (b) as a sole receptor as well as the FRET pair to a stepwise addition (down arrow) and subsequent removal (up arrow) of the indicated concentrations of MeAsp. The data were normalized to the saturated response. Error bars indicate standard deviation of three independent replicates using different cell growth cultures. Wherever invisible, error bars are smaller than the symbol size.

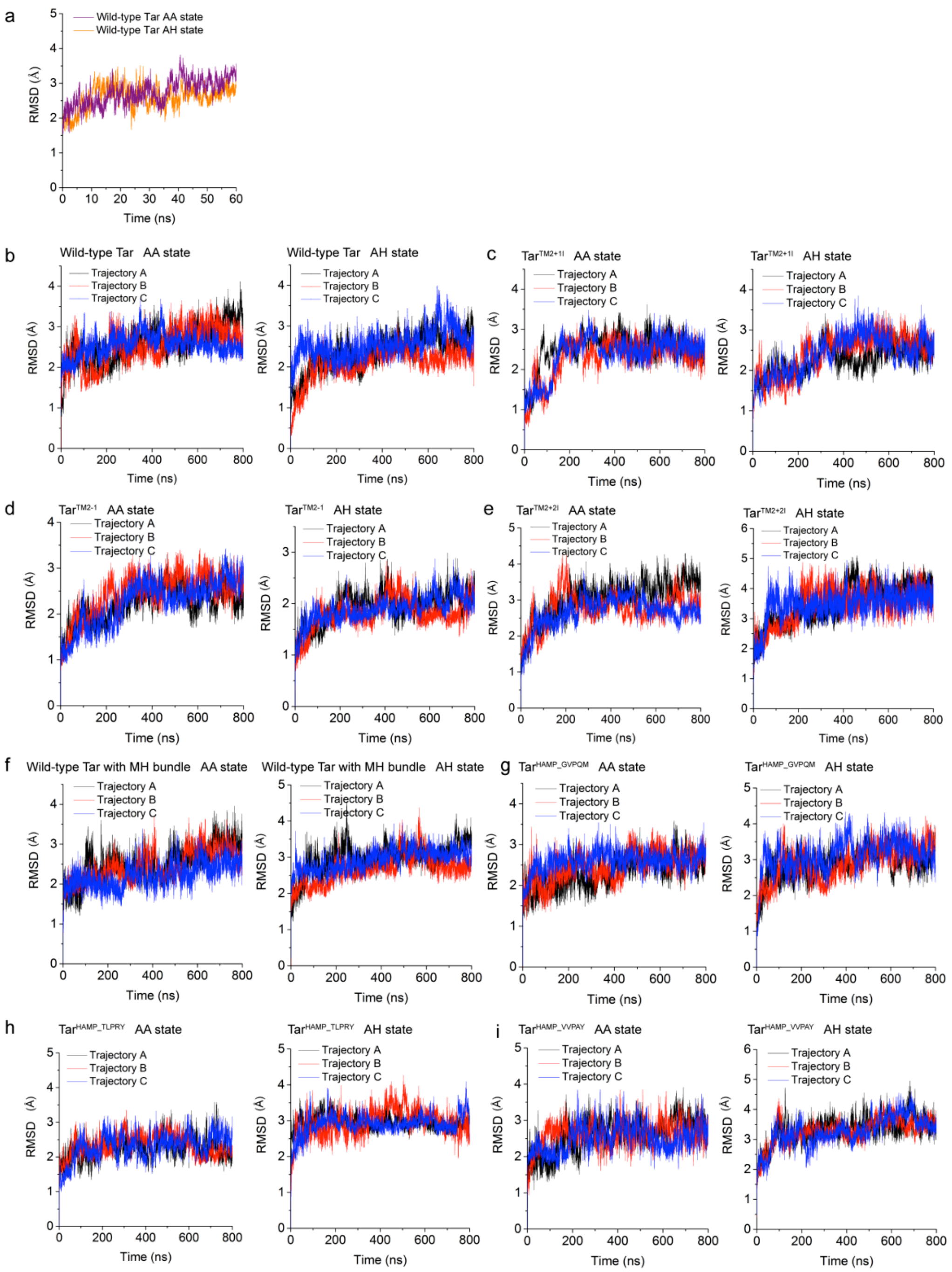

Supplementary Figure 5. Cα RMSD for the AA and AH states of the wild-type and mutant Tar receptors simulated in DPPC at 323 K. (a-i) Cα RMSD from the initial structure as a function of simulation time for (a) final model building stage (see Supplementary Table 8) for the AA and AH states of wild-type Tar with decreased restraints, and (b-i) the three independent 800 ns MD simulations for the AA and AH states of wild-type Tar and indicated mutants.

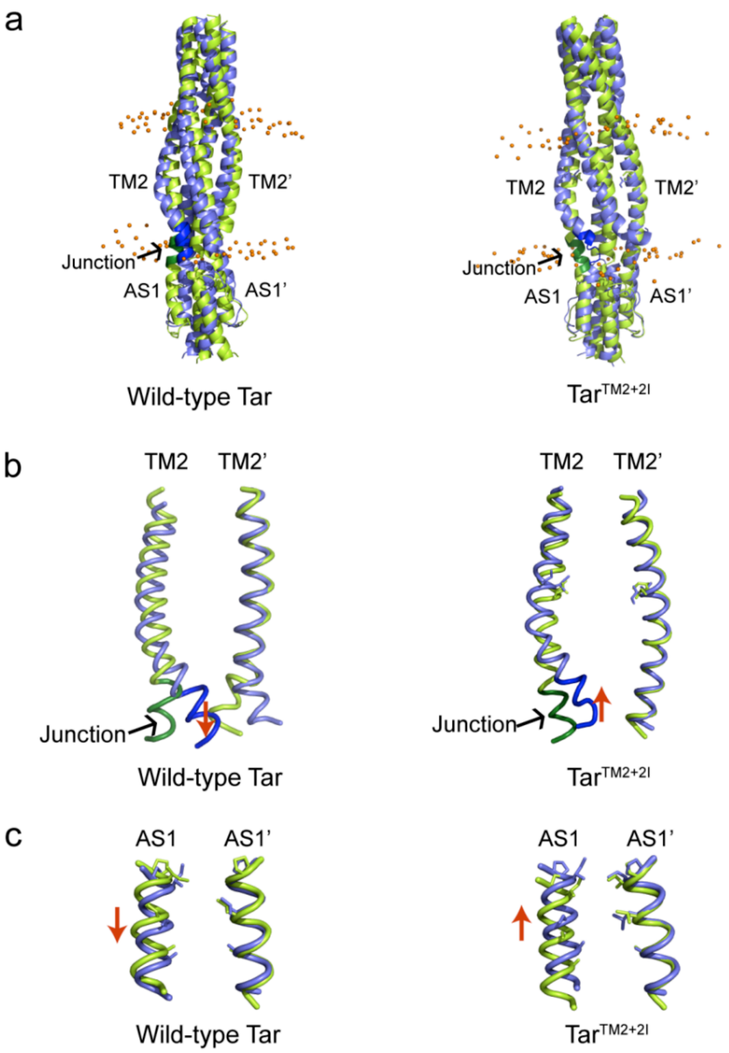

Supplementary Figure 6. Structural differences between AA (green) and AH (blue) models for wild-type Tar and Tar^{TM2+2I} averaged from MD simulations. (a) Structural alignment of AA and AH states for wild-type Tar and Tar^{TM2+2I}. The TM2-AS1 junction residues ^{211}GIRRMLLT^{218} are shown in dark green for AA states and dark blue for AH states, respectively. DPPC phosphorus atoms are represented as orange spheres. In the AH states, TM2 and AS1 are from the ligand-occupied monomer, while TM2' and AS1' are from the ligand-free monomer. (b) Structural alignment of TM2 and TM2' in AA and AH states for wild-type Tar and Tar^{TM2+2I}. The relative sliding of the junction region in the AH state of each receptor is shown by red arrow. In (a,b), the two isoleucines inserted in TM2 are indicated. (c) Structural alignment of AS1 and AS1' in the AA and AH states for wild-type Tar and Tar^{TM2+2I}. The relative sliding of AS1 to AS1' in the AH state is shown by red arrow. The residues on the AS1-AS1' interface are indicated.

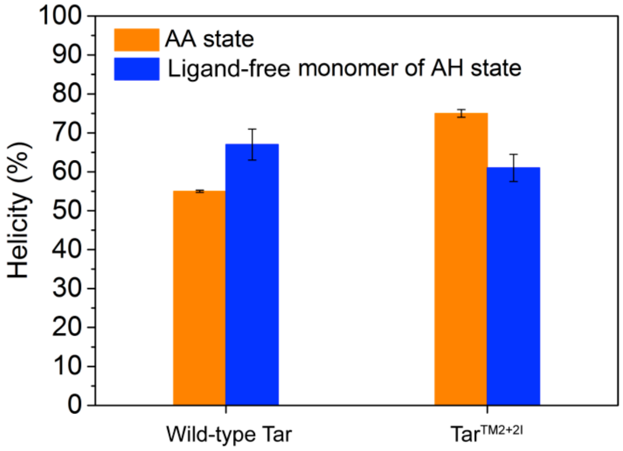

Supplementary Figure 7. Helicity of the junction residues ^{211}GIRRMLLT^{218} in the ligand-free monomer of wild-type Tar or Tar^{TM2+2I}. The helicity of the junction residues was averaged over three independent MD simulations for final 600 ns in DPPC. The values are shown as mean ± standard deviation.

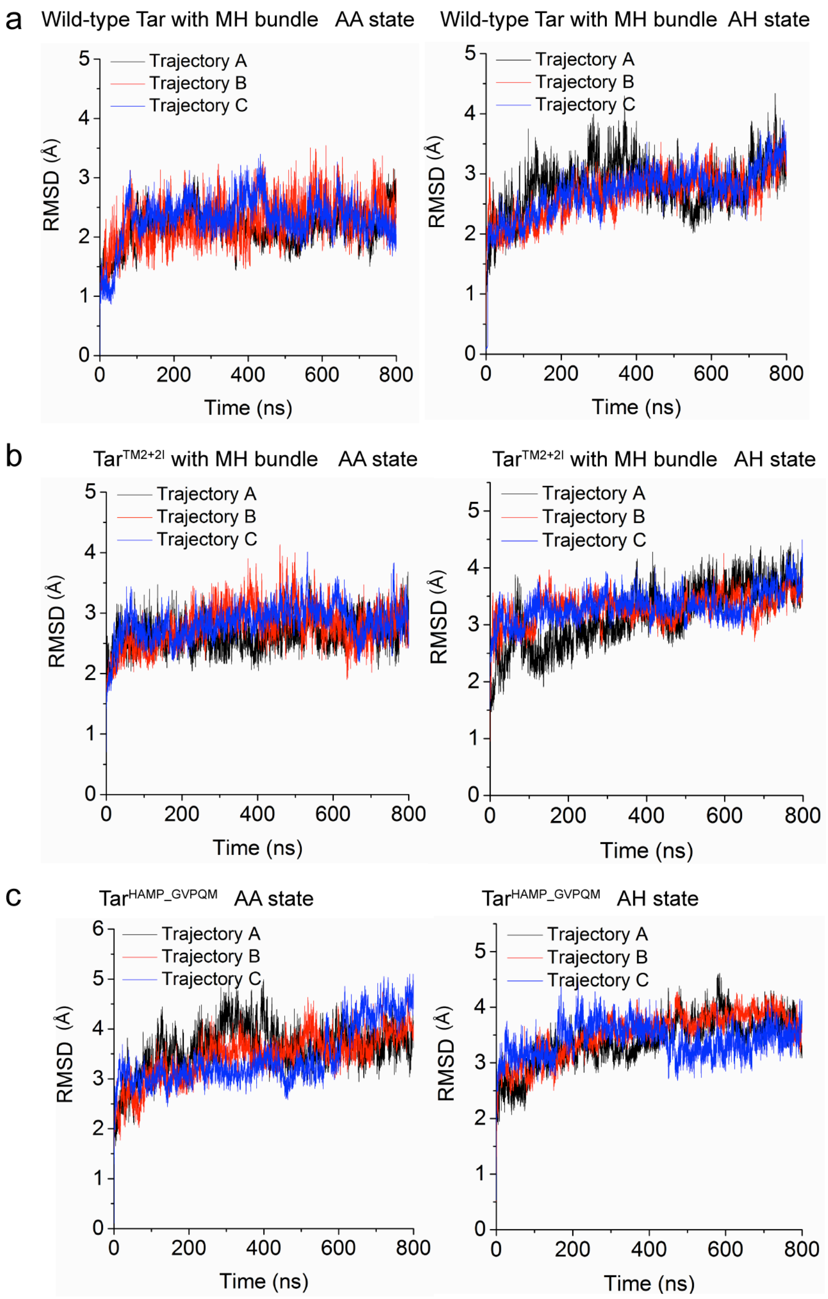

Supplementary Figure 8. Cα RMSD for the AA and AH states of the wild-type and mutant Tar receptors simulated in POPC at 300 K. (a-c) Cα RMSD from the initial structure as a function of time for the three independent MD simulations for the AA and AH states of wild-type Tar and indicated mutants.

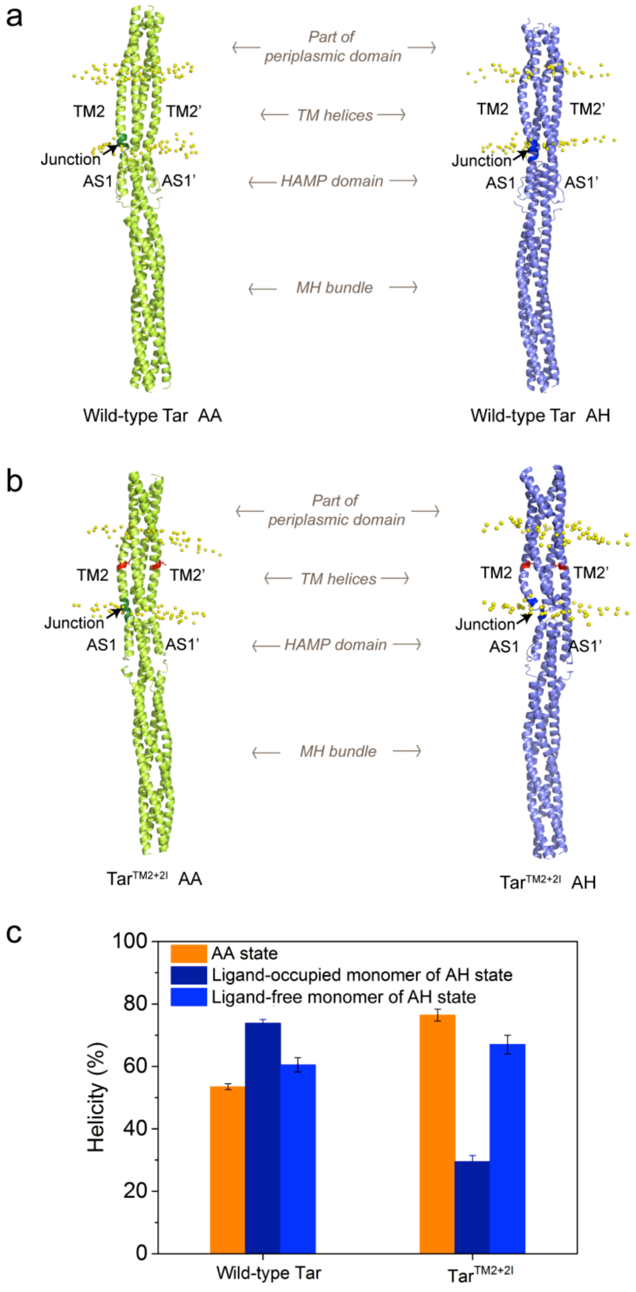

Supplementary Figure 9. MD simulations for the receptor fragment in POPC at 300 K. The simulated fragment contains a part of the periplasmic domain, the transmembrane helices, the HAMP domain, and the MH bundle. (a,b) Representative structures of AA (green) and AH (blue) models for wild-type Tar (a) and Tar^{TM2+2I} (b) averaged from MD simulations. Notations for the helices in the TM and HAMP domains are shown along the structures. In AH states, TM2 and AS1 are from the ligand-occupied monomer, while TM2' and AS1' belong to the ligand-free monomer. Two isoleucines added in TM2 are shown in red in (b). The TM2-AS1 junction residues

^{211}GIRRMLLT^{218} are indicated. POPC phosphorus atoms are represented as yellow spheres to show the solvent-membrane interfaces. The structures were prepared using PyMol. (c) The helicity of TM2-AS1 junction residues ^{211}GIRRMLLT^{218} was averaged over three independent MD simulation trajectories and compared between wild-type Tar and Tar^{TM2+2I}. For the AH state, the helicity of the junction regions of both ligand-occupied monomer and ligand-free monomer was analyzed. The values are shown as mean ± standard deviation.

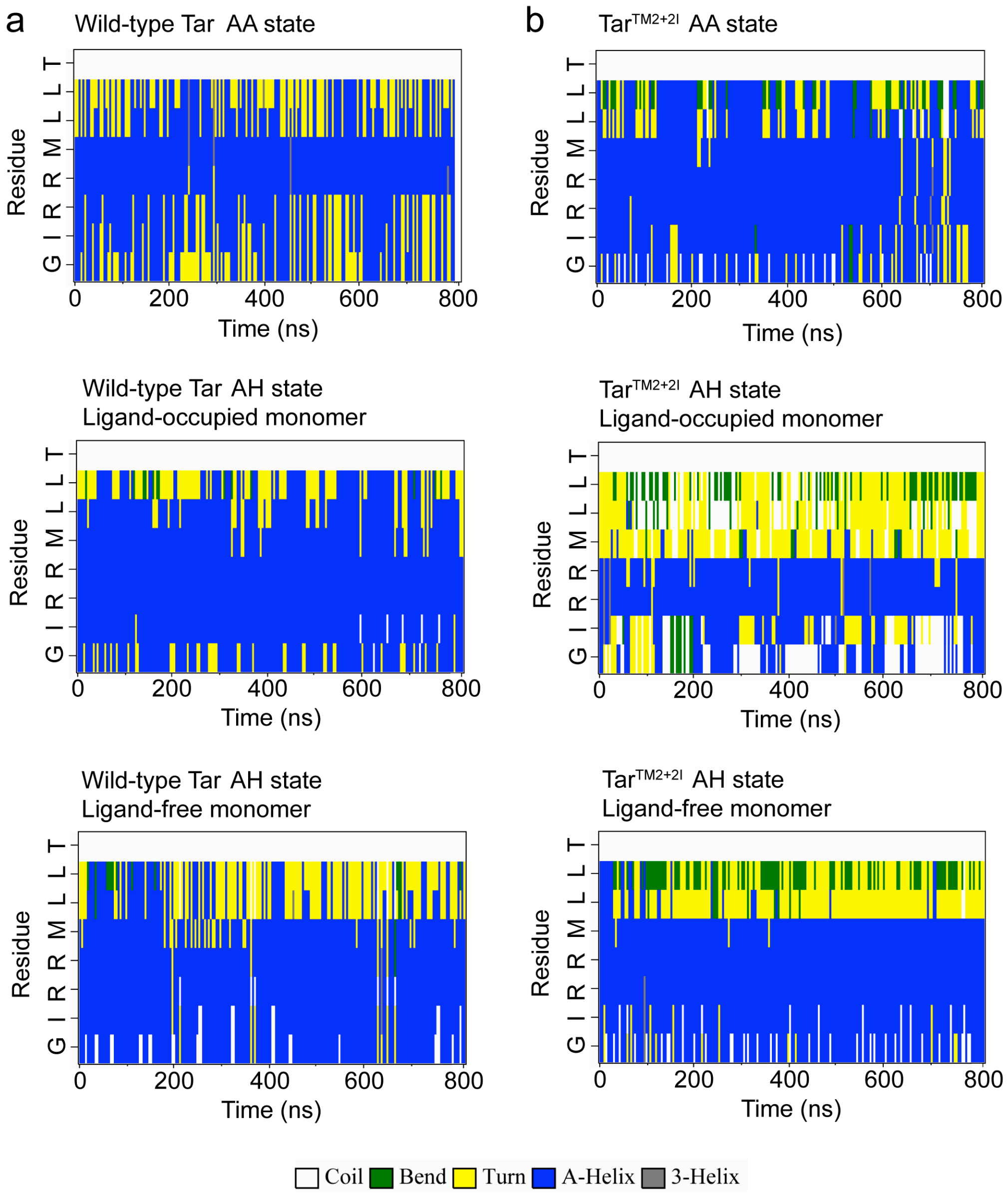

Supplementary Figure 10. Secondary structure profiles of the AA and AH states of wild-type Tar and Tar^{TM2+2I}. The time evolution of secondary structure of the junction residues ^{211}GIRRMLLT^{218} in the AA and AH states (for both ligand-occupied monomer and ligand-free monomer) of wild-type Tar (a) and Tar^{TM2+2I} (b) simulated in DPPC.

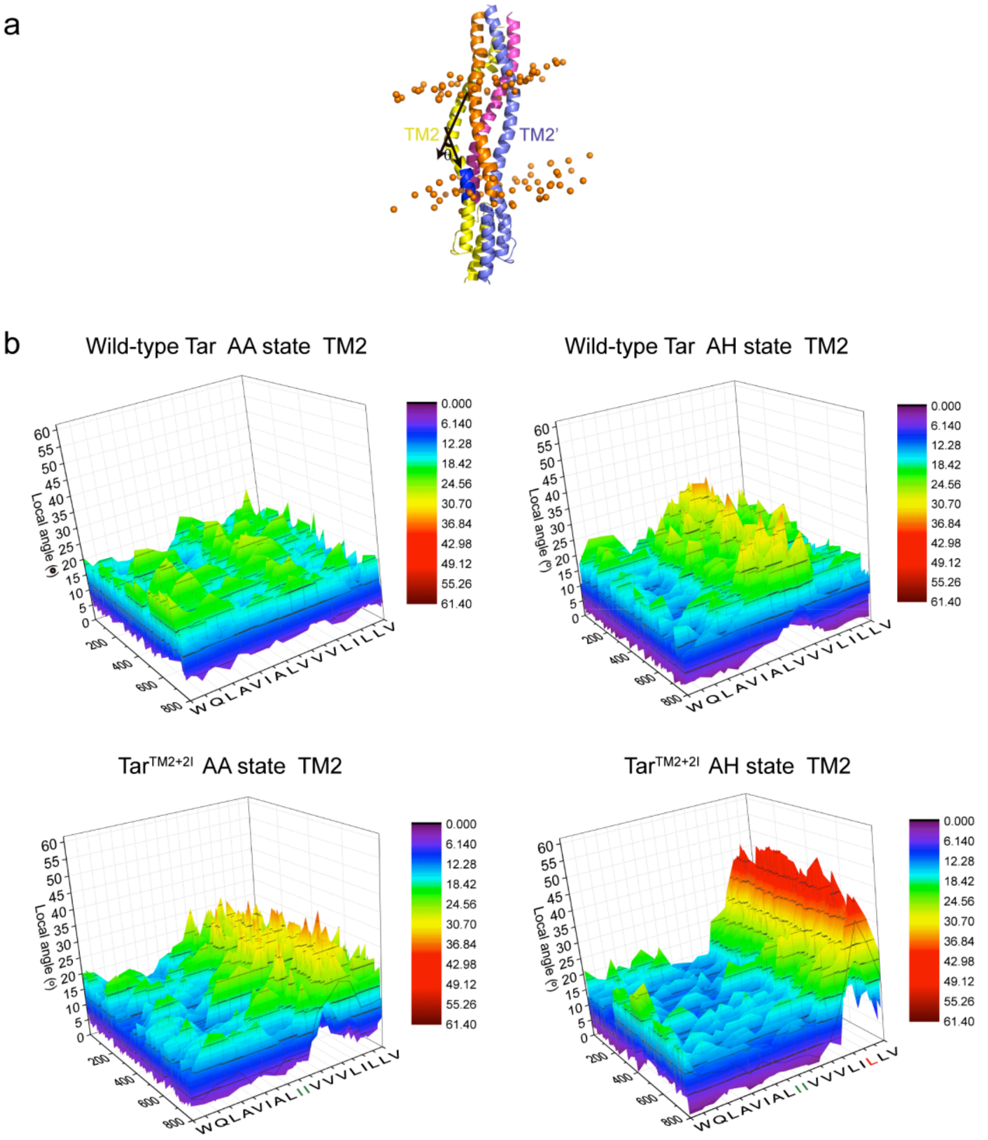

Supplementary Figure 11. Bending angle of TM2 and TM2' in the lipid bilayer and the time evolution of helix curvature profile of TM2 in DPPC lipids. (a) Bending angle θ is defined as the angle between the helical axes of two segments in TM2 or TM2' (Supplementary Table 1, Supplementary Ref. 1). The protein structure is from the MD simulations for Figure 2. (b) The time evolution of helix curvature profile of TM2 residues ^{192}WQLAVIAL(II)VVVLILLV^{207} in the AA and AH states of wild-type Tar and Tar^{TM2+2I} calculated by using the HELANAL module of MDAnalysis. In Tar^{TM2+2I}, insertion of two isoleucines in TM2 is indicated in green. The residue Leu205 is shown in red in the AH state of Tar^{TM2+2I}.

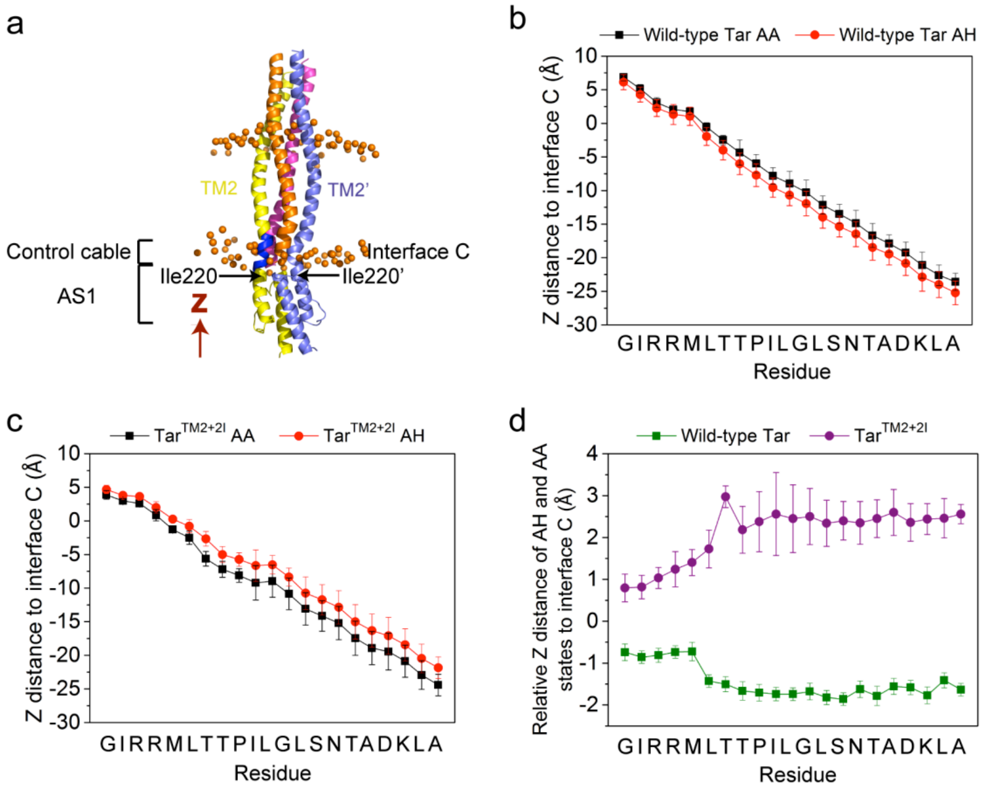

Supplementary Figure 12. The longitudinal distance of the control cable and AS1 to the lipid-cytoplasm interface and the relative longitudinal displacement of Ile220 to Ile220' in the AA and AH states of wild-type Tar and Tar^{TM2+2I}. (a) The distance along the Z-component (red arrow) of the control cable (residues GIRRM) and AS1 (residues LLTPILGLSNTADKLA) to the lipid-cytoplasm interface (mass center of phosphorus atoms in cytoplasmic lipid leaflet; interface C). The relative Z-component of the residue Ile220 to Ile220' near the interface C indicates the longitudinal displacement of AS1 relative to AS1' (Supplementary Table 2). In the AH state, Ile220 is from the ligand-occupied monomer, Ile220' is from the ligand-free monomer. The protein structure is from the MD simulations for Figure 2. (b,c) The Z distance of the control cable and AS1 residues to the center of interface C (zero-point in b and c) in one monomer of the AA and the ligand-occupied monomer of AH states of wild-type Tar (b) and Tar^{TM2+2I} (c). Data were averaged over three independent MD simulations for final 600 ns in DPPC, shown as mean ± standard deviation. (d) The relative Z-component of the control cable and AS1 in the AH and AA states of wild-type Tar and Tar^{TM2+2I} to the interface C.

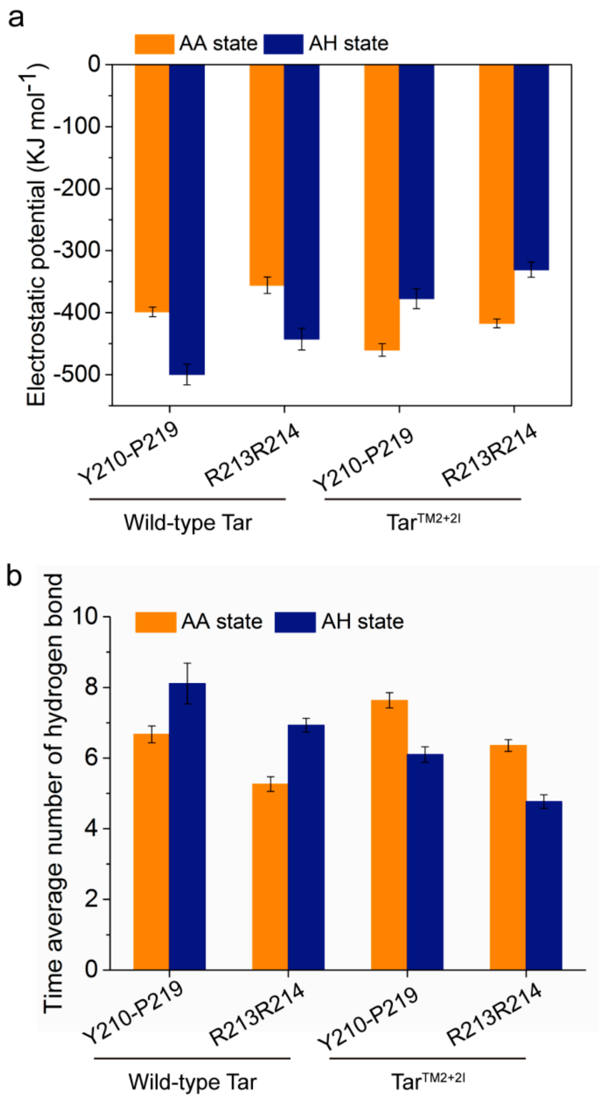

Supplementary Figure 13. The protein-lipid interactions between the residues ^{210}YGIRRMLLTP^{219} and the phospholipids in the AA and AH states of wild-type Tar and Tar^{TM2+2I}. (a) Average electrostatic interactions between the residues ^{210}YGIRRMLLTP^{219} (Y210-P219) or R213R214 and the phospholipids in the AA and AH states of wild-type Tar and Tar^{TM2+2I}. (b) Average number of hydrogen bonds formed between the residues ^{210}YGIRRMLLTP^{219} or R213R214 and the lipids in the AA and AH states of wild-type Tar and Tar^{TM2+2I}. Data were averaged over three independent MD simulations for final 600 ns in DPPC, shown as mean ± standard deviation. The electrostatic interactions and hydrogen bonds formed by one monomer in the AA state and by the ligand-occupied monomer in the AH state were analyzed.

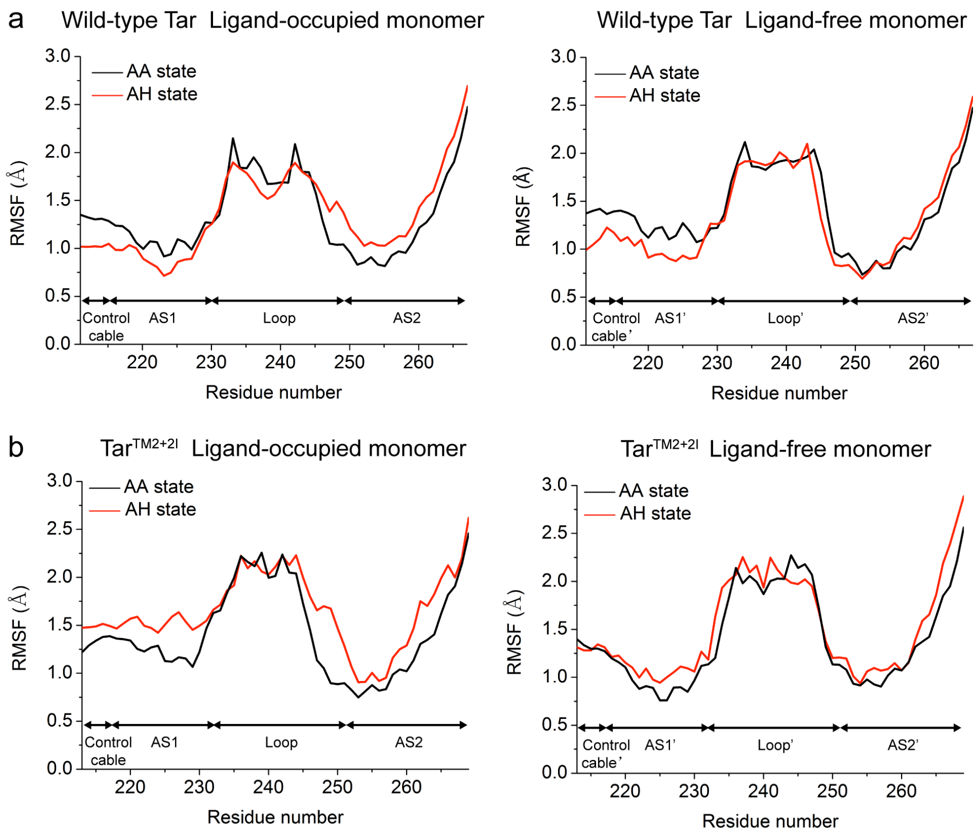

Supplementary Figure 14. RMSF plots of the control cable and the HAMP domain in the AA and AH states simulated in DPPC lipids. The plots for the AA and AH states are shown in black and red, respectively, for the wild-type Tar (a) and Tar^{TM2+2I} (b). Data were averaged over three MD simulation trajectories.

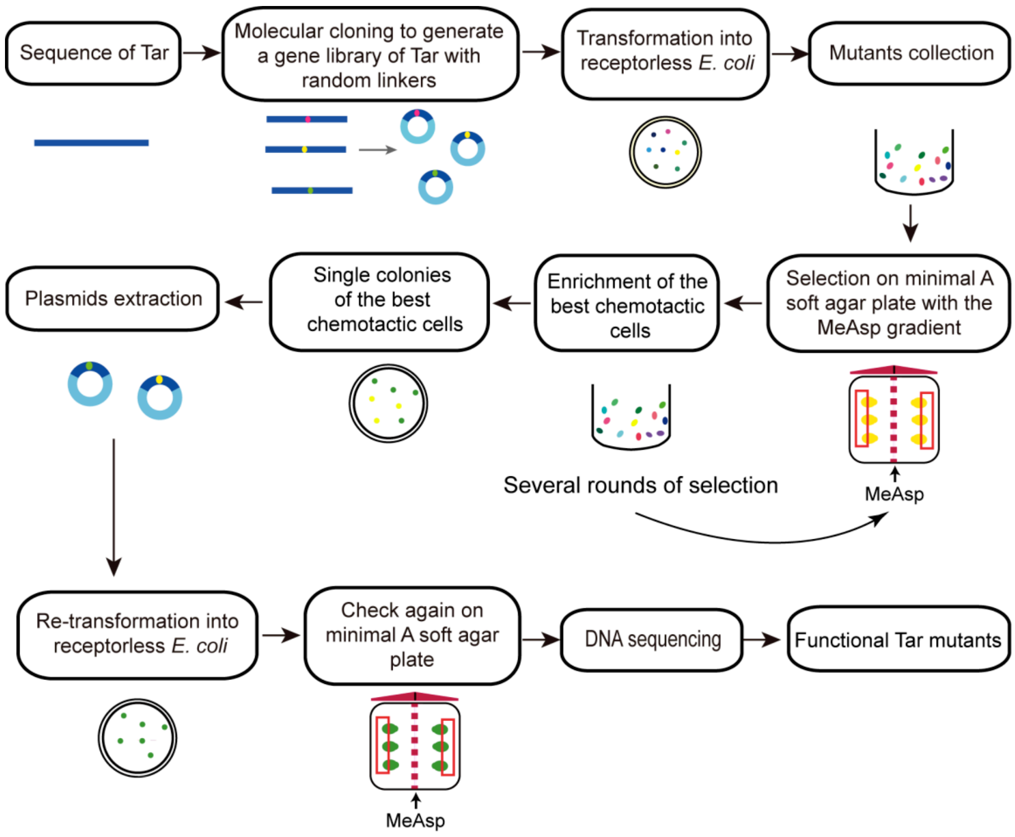

Supplementary Figure 15. Methods for selection of Tar mutants from the Tar[1-266]-XXXXX-[272-553] library. The library of Tar[1-266]-XXXXX-[272-553] was generated by molecular cloning. 10 mM MeAsp solution was applied as a line to the center of the minimal A soft agar plate to generate a chemical gradient. Overnight culture expressing the library was applied to the plate at a defined distance from the center and incubated at 30°C. Cells that migrated the farthest in the chemical gradient were re-inoculated on a new plate for the second round of selection. Receptor-expressing plasmids for the best-chemotactic cells were isolated. The sequence of the linker in the selected receptor mutants was identified by DNA sequencing.

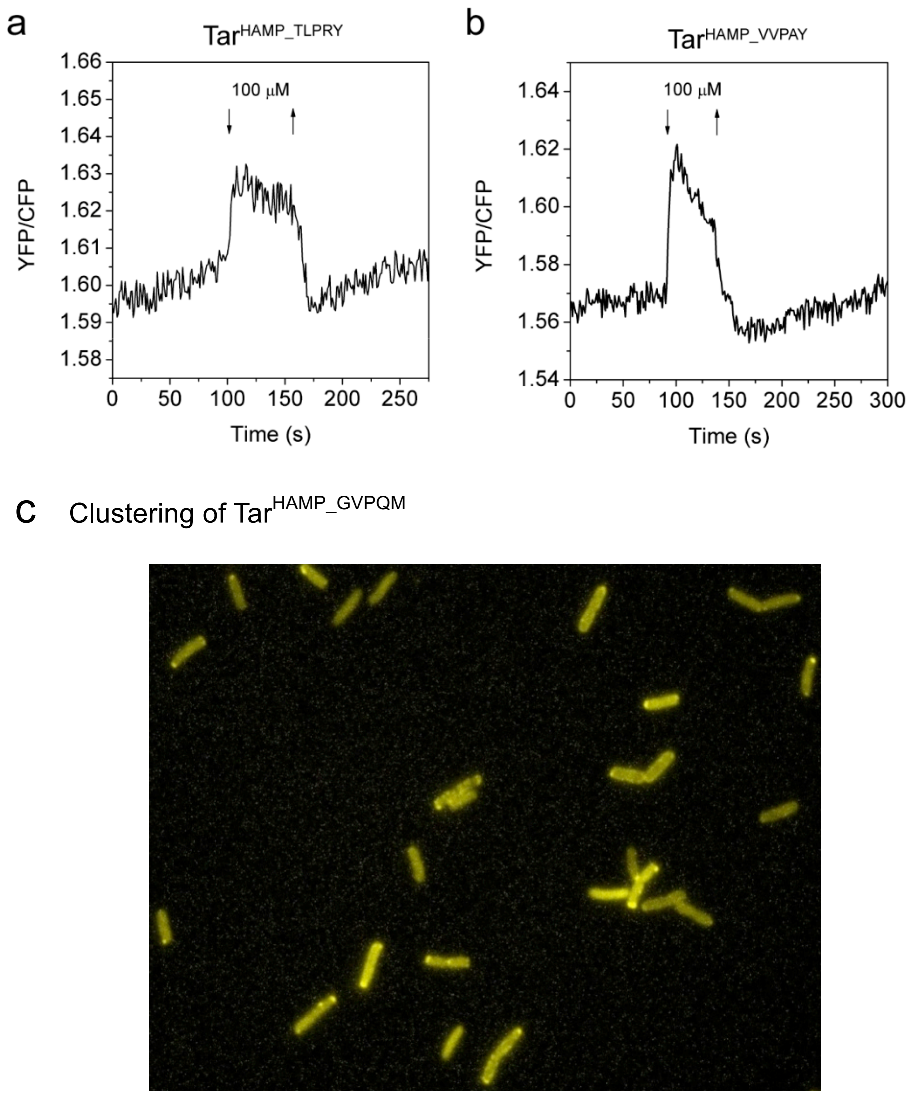

Supplementary Figure 16. FRET responses of Tar^{HAMP_TLPRY} and Tar^{HAMP_VVPAY} to MeAsp and clustering of Tar^{HAMP_GVPQM}. (a,b) Buffer-adapted *E. coli* CheR^{+}CheB^{+} strain VS181 expressing Tar^{HAMP_TLPRY} (a) or Tar^{HAMP_VVPAY} (b) as a sole receptor as well as the FRET pair was stimulated by a stepwise addition (down arrow) and subsequent removal (up arrow) of indicated concentrations of MeAsp. (c) Tar^{HAMP_GVPQM} was co-expressed in receptorless CheR^{+}CheB^{+} strain UU1250 together with catalytically inactive YFP-CheR^{D154A} that marks clusters by binding to receptors.

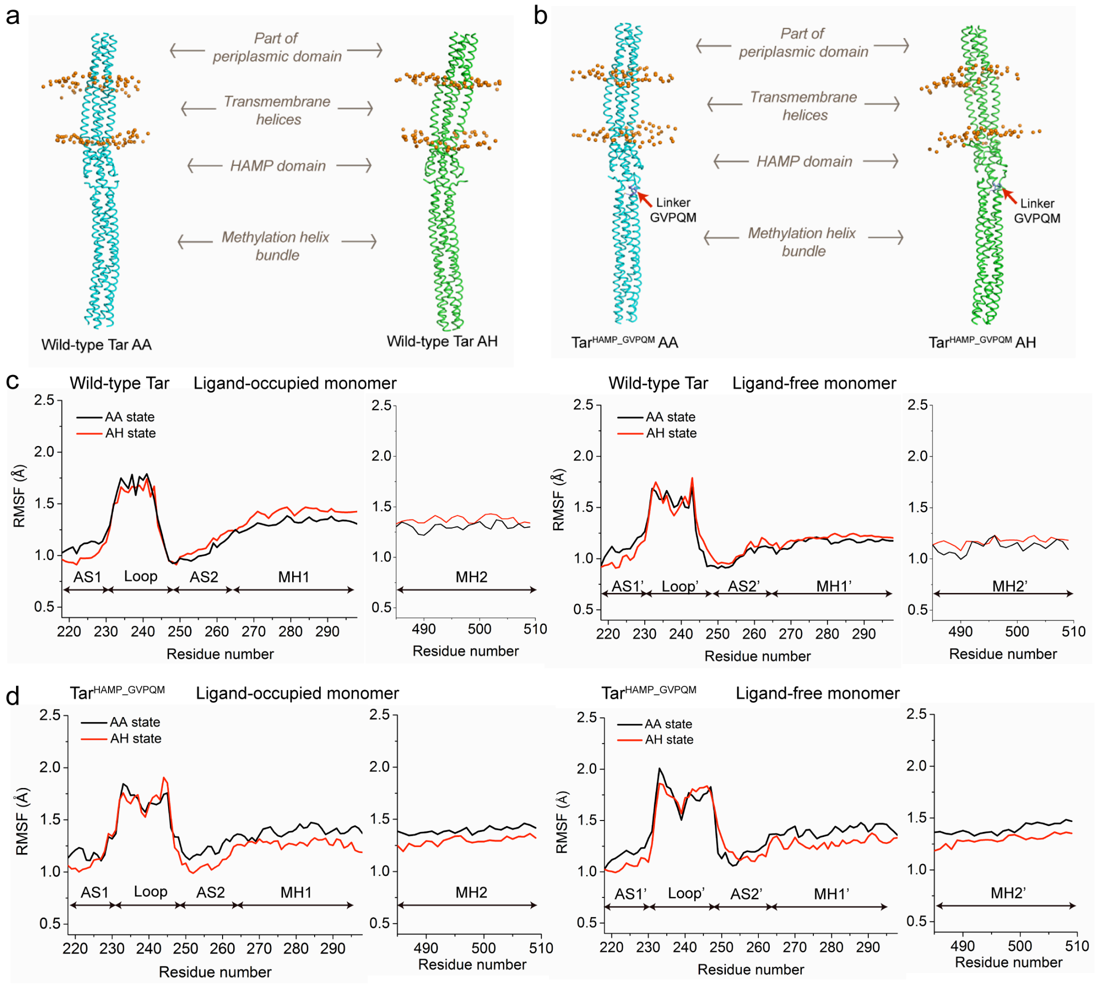

Supplementary Figure 17. The structures and RMSF plots of the AA and AH states of the wild-type Tar and Tar^{HAMP_GVPQM} simulated in DPPC at 323 K. (a,b) Representative structure of AA (cyan) and AH (green) models of wild-type Tar (a) and Tar^{HAMP_GVPQM} (b). The linker region of Tar^{HAMP_GVPQM} is shown in blue. (c,d) RMSF of the AA and AH states of the wild-type Tar (c) and Tar^{HAMP_GVPQM} (d). The plots for the AA and AH states are shown in black and red, respectively. Data were averaged over three MD simulation trajectories.

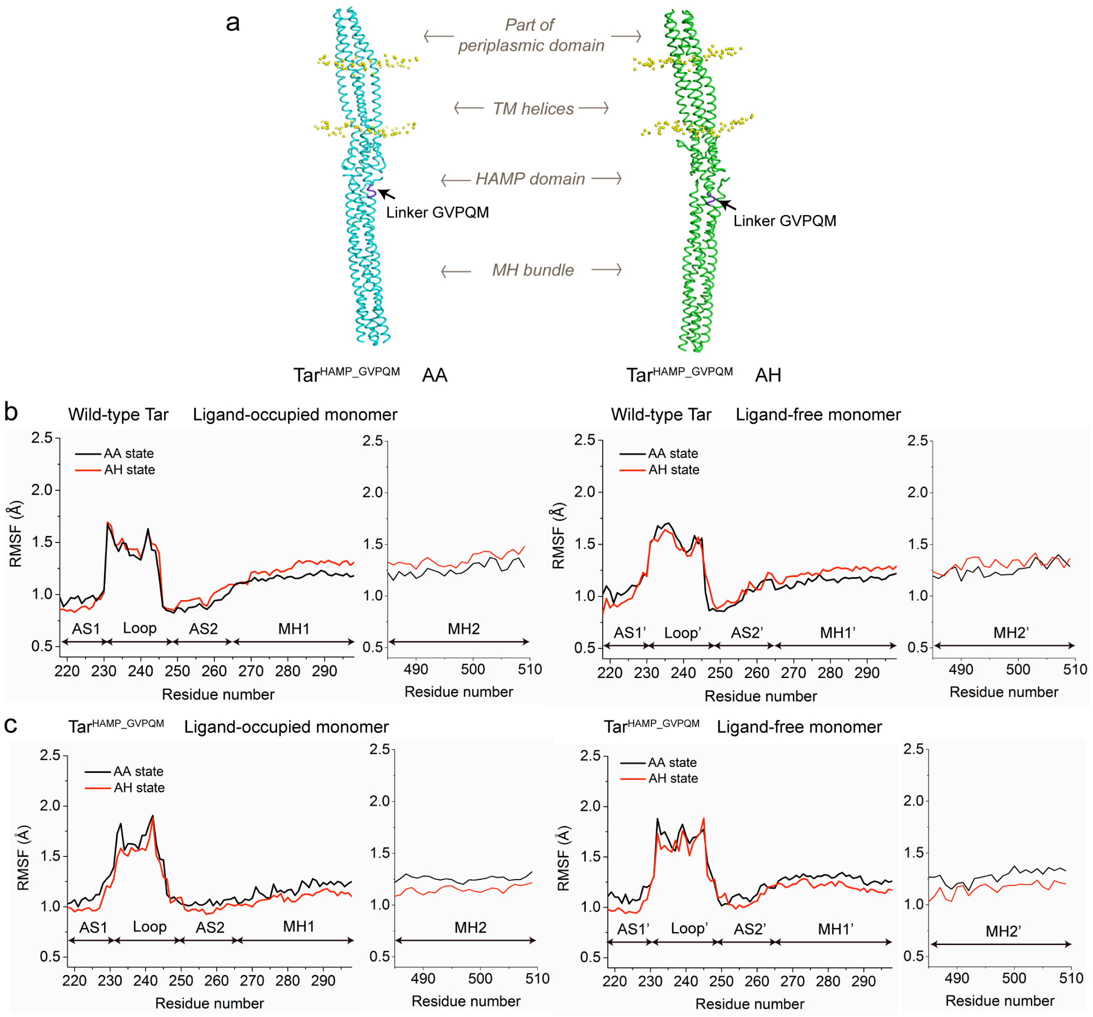

Supplementary Figure 18. The structures and RMSF plots of the AA and AH states of the wild-type and Tar^{HAMP_GVPQM} simulated in POPC at 300 K. (a) Representative structure of AA (cyan) and AH (green) models of Tar^{HAMP_GVPQM}. The linker region of Tar^{HAMP_GVPQM} is shown in blue. (b,c) RMSF of the AA and AH states of the wild-type Tar (b) and Tar^{HAMP_GVPQM} (c). The plots for the AA and AH states are shown in black and red, respectively. Data were averaged over three MD simulation trajectories.

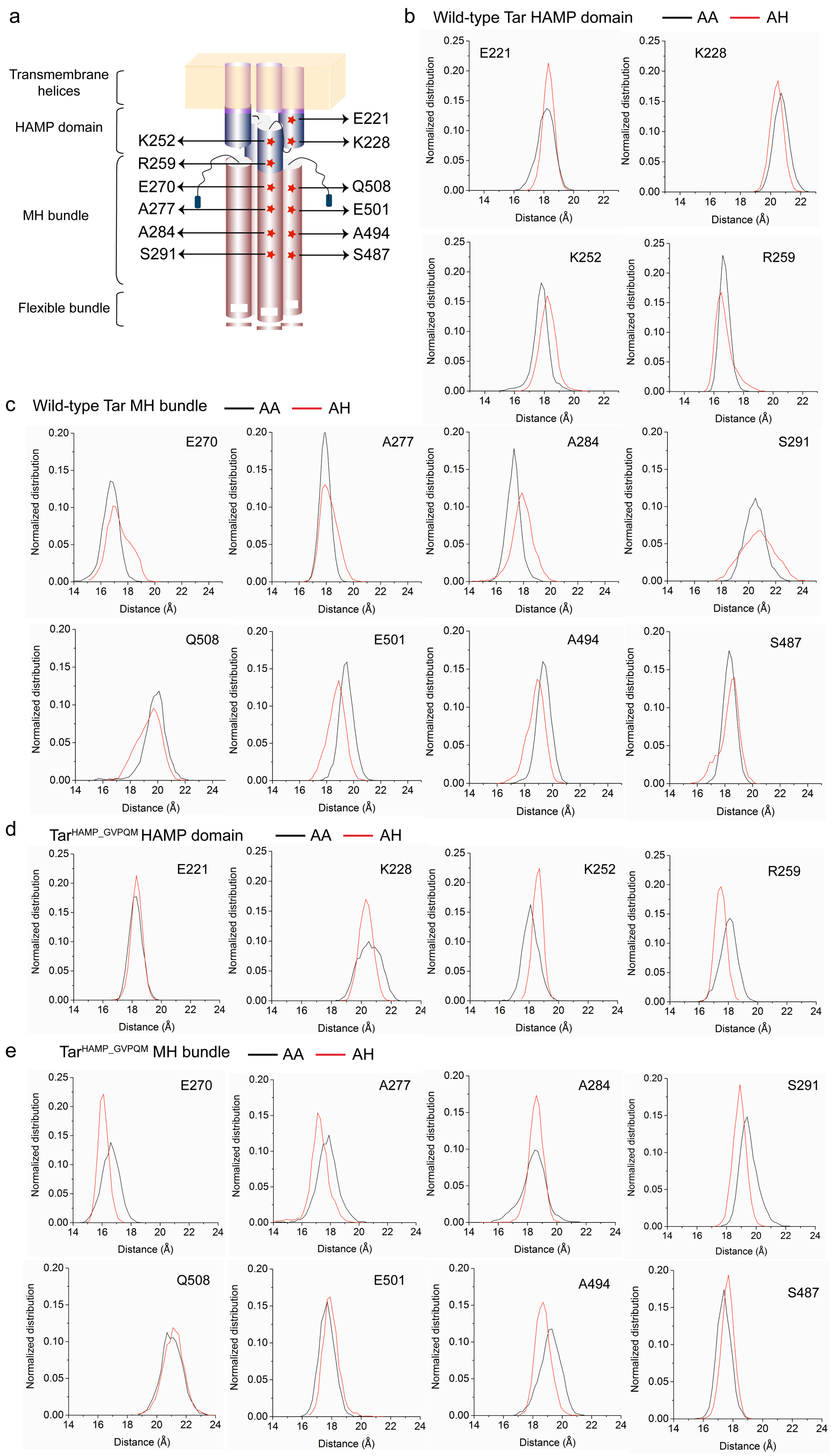
a
Transmembrane helices
HAMP domain
MH bundle
Flexible bundle
E221
K252
K228
R259
E270
Q508
A277
E501
A284
A494
S291
S487
b
Wild-type Tar HAMP domain
AA
AH
c
Wild-type Tar MH bundle
d
TarHAMP_GVPQM HAMP domain
e
TarHAMP_GVPQM MH bundle
Normalized distribution
Distance (Å)

Supplementary Figure 19. Distributions of distances between the residue pairs for the two monomers in the AA and AH states of wild-type Tar and Tar^{HAMP_GVPQM}. (a) The position of the selected residues on the HAMP domain and MH bundle. (b-e) The distance distributions of the selected residue pairs between the two monomers, for example, between E221 and E221'.

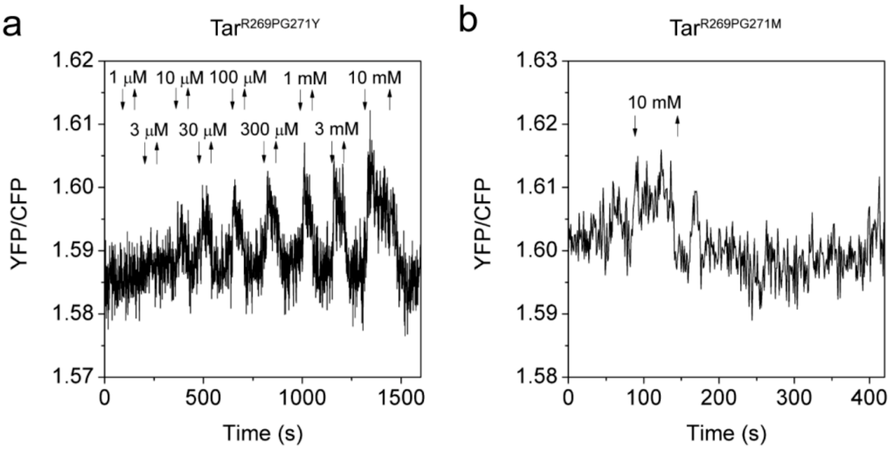

Supplementary Figure 20. FRET responses of Tar^{R269PG271Y} and Tar^{R269PG271M} to MeAsp. (a,b) Buffer-adapted *E. coli* CheR^{+}CheB^{+} strain VS181 expressing Tar^{R269PG271Y} (a) or Tar^{R269PG271M} (b) as a sole receptor as well as the FRET pair was stimulated by a stepwise addition (down arrow) and subsequent removal (up arrow) of indicated concentrations of MeAsp.

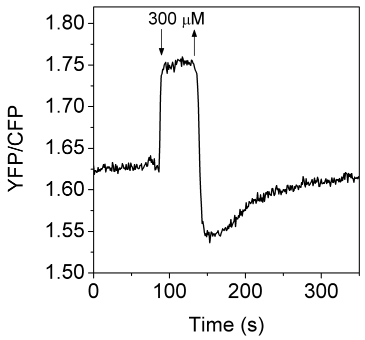

Supplementary Figure 21. Response of strain VS181 expressing Tar°-T303I to $NiCl_2$. See text for details. Buffer-adapted *E. coli* CheR$^+$CheB$^+$ strain VS181 expressing Tar°-T303I as a sole receptor as well as the FRET pair was stimulated by a stepwise addition (down arrow) and subsequent removal (up arrow) of indicated concentration of $NiCl_2$.

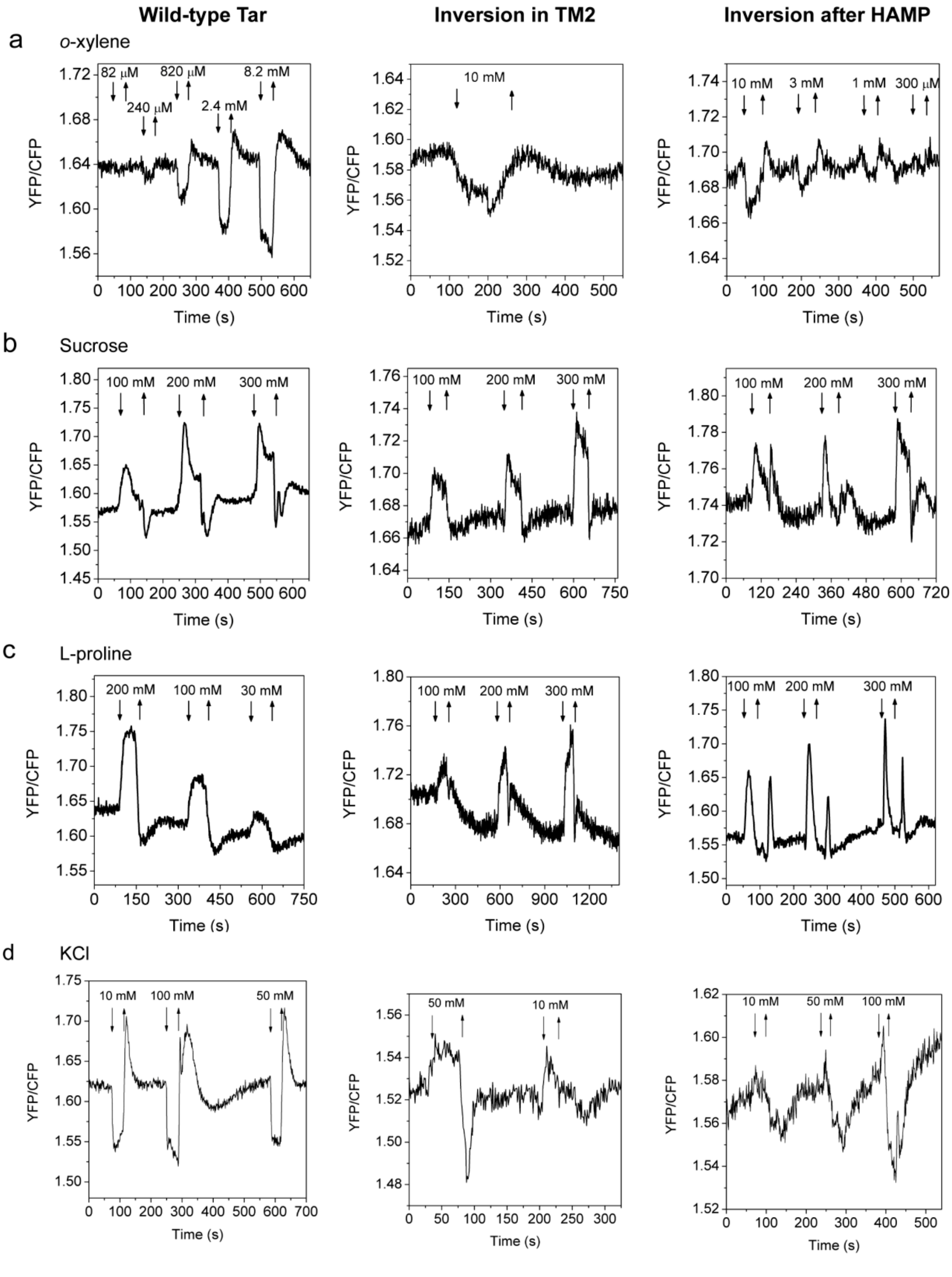

Supplementary Figure 22. Responses of the wild-type and mutant Tar to *o*-xylene, osmotic stress, and KCl. (a-d) FRET responses of buffer-adapted *E. coli* CheR^{+}CheB^{+} strain VS181 expressing the wild-type Tar, Tar^{TM2+2I} (inversion in TM2), and Tar^{HAMP_GVPQM} (inversion after HAMP) as a sole receptor as well as the FRET pair to a stepwise addition (down arrow) and subsequent removal (up arrow) of the indicated concentrations of *o*-xylene (a), sucrose (b), L-proline (c) and KCl (d).

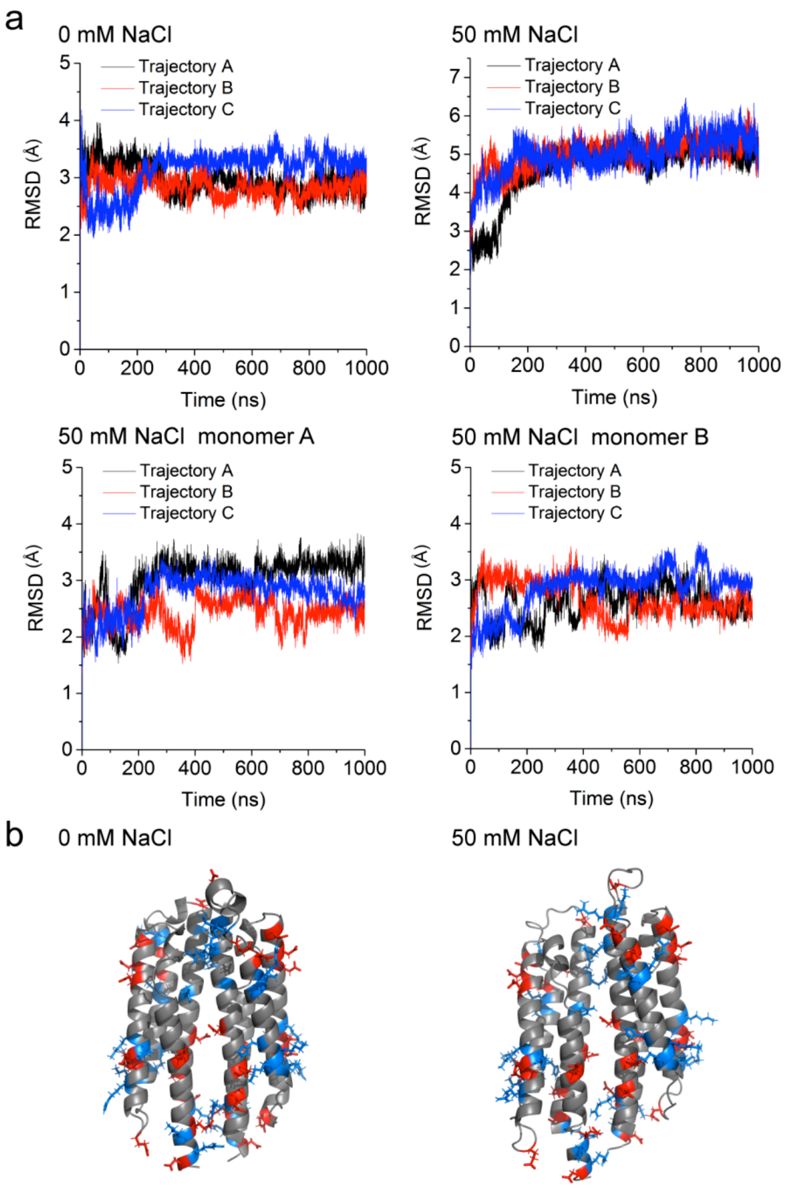

Supplementary Figure 23. Simulated effect of NaCl on the periplasmic domain of Tar. (a) Cα RMSD from the initial structure as a function of simulation time for the three independent 1000 ns MD simulations for the Tar periplasmic domain at 0 or 50 mM NaCl. The average RMSD for the dimer of Tar periplasmic domain at 50 mM NaCl is larger due to the relative motion of the two monomers. The RMSD for the individual monomer at 50 mM NaCl remains around 3 Å. (b) Structure of the periplasmic domain was simulated either at 0 or 50 mM NaCl. Positively charged residues are shown in blue and negatively charged residues are shown in red.

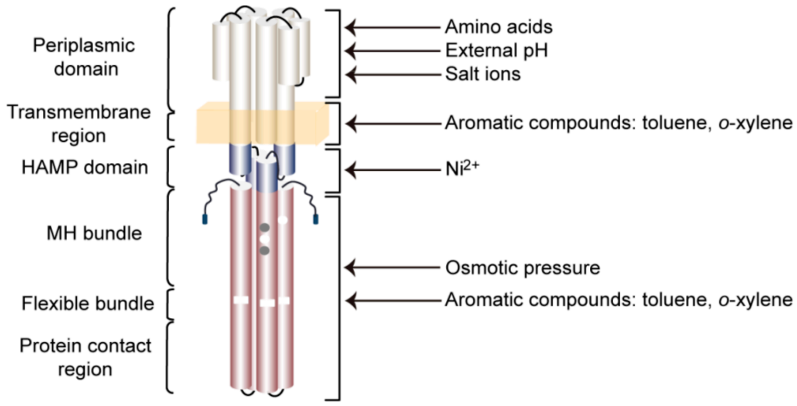

Supplementary Figure 24. Regions of Tar responsible for detection of environmental stimuli. See text for details. Schematic representation of domain structure of Tar, with regions for sensing amino acids, external pH, salt ions, aromatic compounds, Ni^{2+}, and osmotic pressure being indicated.

Supplementary Tables

Supplementary Table 1. Bending angle of TM2 and TM2' calculated from the MD simulations

Receptor	Simulation trajectory[a]	AA (°)		AH (°)	
		TM2'	TM2	TM2'[b]	TM2[c]
**Simulation in DPPC at 323 K**					
Wild-type Tar	A	20.1 ± 3.2[d]	17.5 ± 3.4	25.8 ± 3.7	33.8 ± 2.5
Wild-type Tar	B	25.8 ± 2.9[d]	22.8 ± 1.9	26.9 ± 2.5	37.1 ± 3.2
Wild-type Tar	C	22.9 ± 2.1[d]	24.7 ± 2.2	23.7 ± 2.2	32.7 ± 3.9
Wild-type Tar	Average	22.9 ± 2.9[e]	21.7 ± 3.7	25.5 ± 1.6	34.5 ± 2.3
Tar^{TM2+2I}	A	28.2 ± 3.7	30.1 ± 3.5	15.8 ± 4.8	51.6 ± 4.6
Tar^{TM2+2I}	B	31.1 ± 4.5	32.8 ± 4.1	10.7 ± 4.2	51.0 ± 3.1
Tar^{TM2+2I}	C	29.1 ± 3.4	31.8 ± 3.6	10.4 ± 3.5	49.5 ± 3.5
Tar^{TM2+2I}	Average	29.5 ± 1.5	31.6 ± 1.4	12.3 ± 3.0	50.7 ± 1.1
Tar^{TM2+1I}	A	21.1 ± 3.9	26.0 ± 3.4	29.3 ± 2.1	33.1 ± 3.8
Tar^{TM2+1I}	B	24.9 ± 3.1	24.5 ± 2.9	26.8 ± 3.5	37.3 ± 2.8
Tar^{TM2+1I}	C	24.5 ± 2.5	27.8 ± 2.5	25.4 ± 2.4	38.2 ± 2.8
Tar^{TM2+1I}	Average	23.5 ± 2.1	26.1 ± 1.7	27.1 ± 2.0	36.1 ± 2.7
Tar^{TM2-1}	A	18.6 ± 2.9	19.8 ± 3.9	20.7 ± 2.4	30.4 ± 3.1
Tar^{TM2-1}	B	19.1 ± 2.4	21.3 ± 2.8	25.9 ± 2.3	33.9 ± 2.1
Tar^{TM2-1}	C	22.4 ± 3.1	22.4 ± 2.2	24.9 ± 2.1	31.6 ± 2.2
Tar^{TM2-1}	Average	20.0 ± 2.1	21.2 ± 1.3	23.8 ± 2.8	32.0 ± 1.8
**Simulation in POPC at 300 K**					
Wild-type Tar	A	21.8 ± 2.2	23.9 ± 1.9	25.2 ± 2.1	33.8 ± 1.8
Wild-type Tar	B	19.1 ± 1.8	24.5 ± 2.1	22.6 ± 2.6	36.1 ± 3.0
Wild-type Tar	C	20.6 ± 2.5	21.1 ± 1.7	23.4 ± 2.9	37.2 ± 2.6
Wild-type Tar	Average	20.5 ± 1.4	23.2 ± 1.8	23.7 ± 1.3	35.7 ± 1.7
Tar^{TM2+2I}	A	34.7 ± 2.4	27.7 ±3.1	16.5 ±2.1	46.8 ± 2.6

Tar^{TM2+2I}	B	33.1 ± 1.8	30.1 ±2.8	14.2 ± 3.2	50.6 ± 1.8
Tar^{TM2+2I}	C	31.2 ± 2.5	28.8 ± 2.5	16.7 ± 2.8	45.1 ± 2.1
Tar^{TM2+2I}	Average	33.0 ± 1.8	28.9 ± 1.2	15.8 ± 1.4	47.5 ± 2.8

[a]The trajectories A, B and C for each genetic variant are from three independent MD simulations.

[b,c]In AH state, TM2 is from the ligand-occupied monomer, TM2' is from the ligand-free monomer.

[d]Bending angle is averaged over three independent MD simulations for final 600 ns, shown as mean ± standard deviation.

[e]The averaged bending angle for each genetic variant is the mean of the bending angle calculated from the three trajectories A, B and C, shown as mean ± standard deviation.

Supplementary Table 2. Average longitudinal displacement of Ile220 relative to Ile 220' calculated from the MD simulations

Receptor	Simulation trajectory[a]	Relative displacement[b] (Å)	
		AA	AH
**Simulation in DPPC at 323 K**			
Wild-type Tar	A	-0.16 ± 0.31[c]	-1.79 ± 0.38
Wild-type Tar	B	0.26 ± 0.24[c]	-1.87 ± 0.44
Wild-type Tar	C	0.19 ± 0.31[c]	-1.57 ± 0.23
Wild-type Tar	Average	0.1 ± 0.23[d]	-1.74 ± 0.16
Tar^{TM2+2I}	A	-0.1 ± 0.30	1.49 ± 0.63
Tar^{TM2+2I}	B	0.15 ± 0.22	3.46 ± 0.55
Tar^{TM2+2I}	C	0.22 ± 0.27	2.73 ± 0.54
Tar^{TM2+2I}	Average	0.09 ± 0.17	2.56 ± 0.99
Tar^{TM2+1I}	A	0.26 ± 0.54	-1.88 ± 0.45
Tar^{TM2+1I}	B	-0.15 ± 0.43	-1.55 ± 0.31
Tar^{TM2+1I}	C	0.23 ± 0.42	-1.45 ± 0.38
Tar^{TM2+1I}	Average	0.11 ± 0.23	-1.62 ± 0.22
Tar^{TM2-1}	A	0.32 ± 0.30	-1.54 ± 0.4
Tar^{TM2-1}	B	-0.24 ± 0.31	-1.66 ± 0.32
Tar^{TM2-1}	C	0.29 ± 0.25	-1.73 ± 0.31
Tar^{TM2-1}	Average	0.13 ± 0.32	-1.64 ± 0.10
**Simulation in POPC at 300 K**			
Wild-type Tar	A	-0.10 ± 0.18	-1.58 ± 0.31
Wild-type Tar	B	0.06 ± 0.23	-1.71 ± 0.32
Wild-type Tar	C	-0.08 ± 0.19	-1.66 ± 0.25
Wild-type Tar	Average	-0.04 ± 0.09	-1.65 ± 0.07
Tar^{TM2+2I}	A	0.15 ± 0.22	3.13 ± 0.14
Tar^{TM2+2I}	B	-0.17 ± 0.10	2.24 ± 0.20
Tar^{TM2+2I}	C	0.19 ± 0.19	2.61 ± 0.19
Tar^{TM2+2I}	Average	0.06 ± 0.20	2.64 ± 0.37

[a]The trajectories A, B and C for each genetic variant are from three independent MD simulations.

[b]Longitudinal displacement of Ile220 relative to Ile220' near the lipid-cytoplasm interface. In the AH state, Ile220 is from the ligand-occupied monomer, Ile220' is from the ligand-free monomer.

[c]Longitudinal displacement is averaged over three independent MD simulations for final 600 ns, shown as mean ± standard deviation. A negative displacement corresponds to a piston movement toward the cytoplasmic side.

[d]The averaged longitudinal displacement for each genetic variant is the mean of the longitudinal displacement calculated from the three trajectories A, B and C, shown as mean ± standard deviation.

Supplementary Table 3. Structural dynamics of the control cable and HAMP domain in the AA and AH states of the wild-type Tar and Tar^{TM2+2I} calculated from the simulations using DPPC

Receptor	Control cable[a]		AS1[b]		AS2[c]	
	AA (Å)	AH (Å)	AA (Å)	AH (Å)	AA (Å)	AH (Å)
Wild-type Tar	1.29 ± 0.16	1.14 ± 0.22	1.11 ± 0.20	0.95 ± 0.23	0.91 ± 0.16	1.01 ± 0.20
Tar^{TM2+2I}	1.23 ± 0.23	1.35 ± 0.21	1.02 ± 0.14	1.25 ± 0.19	1.01 ± 0.15	1.04 ± 0.18

[a-c]The values are averaged from the RMSF plots of the residues 211-215 of the control cable and control cable'[a], 216-230 of AS1 and AS1'[b], and 249-260 of AS2 and AS2'[c] over three independent MD simulations for final 600 ns, shown as mean ± standard deviation.

Supplementary Table 4. Structural dynamics of the HAMP domain in the AA and AH states of the wild-type Tar, Tar^{HAMP_GVPQM}, Tar^{HAMP_TLPRY}, and Tar^{HAMP_VVPAY}

Receptor	RMSF of AS1 and AS1'[a]		RMSF of AS2 and AS2'[b]	
	AA (Å)	AH (Å)	AA (Å)	AH (Å)
**Simulation in DPPC at 323 K**				
Wild-type Tar	1.02 ± 0.21	0.91 ± 0.17	0.94 ± 0.18	1.03 ± 0.16
Tar^{HAMP_GVPQM}	1.18 ± 0.15	1.06 ± 0.11	1.19 ± 0.16	1.13 ± 0.15
Tar^{HAMP_TLPRY}	1.19 ± 0.20	1.08 ± 0.23	1.16 ± 0.20	1.11 ± 0.18
Tar^{HAMP_VVPAY}	1.21 ± 0.19	1.13 ± 0.15	1.20 ± 0.15	1.14 ± 0.14
**Simulation in POPC at 300 K**				
Wild-type Tar	1.08 ± 0.25	0.95 ± 0.22	0.98 ± 0.24	1.04 ± 0.20
Tar^{HAMP_GVPQM}	1.15 ± 0.23	1.01 ± 0.18	1.10 ± 0.16	1.02 ± 0.19

[a]The values are averaged from the RMSF plots of the residues 218-230 of AS1 and AS1' of the HAMP dimer over three independent MD simulations for final 600 ns, shown as mean ± standard deviation.

[b]The values are averaged from the RMSF plots of the residues 249-262 of AS2 and AS2' in three MD simulation trajectories.

Supplementary Table 5. Structural dynamics of the MH bundle in the AA and AH states of the wild-type Tar and Tar$^{\text{HAMP_GVPQM}}$ calculated from the simulations using POPC at 300 K

Receptor	RMSF of MH bundle[a]	
	AA (Å)	AH (Å)
Wild-type Tar	1.22 ± 0.17	1.37 ± 0.22
Tar$^{\text{HAMP_GVPQM}}$	1.30 ± 0.18	1.21 ± 0.15

[a]The values were averaged from the RMSF plots of the residues 267-297 of MH1 and MH1' and the residues 485-509 of MH2 and MH2' for the four-helix MH bundle over three independent MD simulations for final 600 ns. The values are shown as mean ± standard deviation.

Supplementary Table 6. Plasmids and *E. coli* strains used in this study

	Genotype or phenotype	Induction	Source or reference
Plasmids			
pKG116	Expression vector, Cam^r	-	[2]
pVS88	CheY-YFP / CheZ-CFP expression plasmid, Amp^r	50 µM IPTG	[3]
pOB30	GFP expression plasmid, Amp^r	100 µM IPTG	[4]
pPA791	TarΔ(44-183)/T303I expression plasmid, Cam^r	2 µM sodium salicylate	[5]
pVS412	YFP-$CheR^{D154A}$ expression plasmid, Amp^r	50 µM IPTG	[6]
pSB13	Tar expression plasmid, pKG116 derivative, T^{768} was mutated to C^{768} to remove the *Nde*I restriction site	2 µM sodium salicylate	This study
pSB14	Tar mutant Tar^{TM2-2} expression plasmid, pKG116 derivative	2 µM sodium salicylate	This study
pSB15	Tar mutant Tar^{TM2-1} expression plasmid, pKG116 derivative	2 µM sodium salicylate	This study
pSB16	Tar mutant Tar^{TM2+1I} expression plasmid, pKG116 derivative	2 µM sodium salicylate	This study
pSB17	Tar mutant Tar^{TM2+2I} expression plasmid, pKG116 derivative	2 µM sodium salicylate	This study
pSB18	Tar mutant Tar^{TM2+3I} expression plasmid, pKG116 derivative	2 µM sodium salicylate	This study
pSB19	Tar mutant Tar^{TM2+2A} expression plasmid, pKG116 derivative	2 µM sodium salicylate	This study
pSB20	Tar mutant $Tar^{V200IV201I}$ expression plasmid, pKG116 derivative	2 µM sodium salicylate	This study
pSB21	Tar mutant Tar^{HAMP_GVPQM} expression plasmid, pKG116 derivative	2 µM sodium salicylate	This study
pSB22	Tar mutant Tar^{HAMP_TLPRY} expression plasmid, pKG116 derivative	2 µM sodium salicylate	This study
pSB23	Tar mutant Tar^{HAMP_VVPAY} expression plasmid, pKG116 derivative	2 µM sodium salicylate	This study
Strains			
UU1250	RP437 Δ *aer* Δ *tsr* Δ (*tar-tap*) Δ *trg*	-	[7]
VS181	RP437 Δ (*cheYcheZ*) Δ *aer* Δ *tsr* Δ (*tar-tap*) Δ *trg*	-	[3]
VH1	RP437 Δ (*cheRcheB*) Δ (*cheYcheZ*) Δ *aer* Δ *tsr* Δ (*tar-tap*) Δ *trg*	-	[8]

Supplementary Table 7. Simulations performed in this work

Simulation system	Lipids	Receptor	State	Number of trajectories	Time length (ns)
A part of periplasmic domain,transmembrane helices, and HAMP domain	DPPC	Wild-type Tar	AA	3	800
		Wild-type Tar	AH	3	800
		Tar^{TM2+2I}	AA	3	800
		Tar^{TM2+2I}	AH	3	800
		Tar^{TM2+1I}	AA	3	800
		Tar^{TM2+1I}	AH	3	800
		Tar^{TM2-1}	AA	3	800
		Tar^{TM2-1}	AH	3	800
A part of periplasmic domain,transmembrane helices, HAMP domain, and MH bundle	DPPC	Wild-type Tar	AA	3	800
		Wild-type Tar	AH	3	800
		Tar^{HAMP_GVPQM}	AA	3	800
		Tar^{HAMP_GVPQM}	AH	3	800
		Tar^{HAMP_TLPRY}	AA	3	800
		Tar^{HAMP_TLPRY}	AH	3	800
		Tar^{HAMP_VVPAY}	AA	3	800
		Tar^{HAMP_VVPAY}	AH	3	800
A part of periplasmic domain,transmembrane helices, HAMP domain, and MH bundle	POPC	Wild-type Tar	AA	3	800
		Wild-type Tar	AH	3	800
		Tar^{TM2+2I}	AA	3	800
		Tar^{TM2+2I}	AH	3	800
		Tar^{HAMP_GVPQM}	AA	3	800
		Tar^{HAMP_GVPQM}	AH	3	800
Periplasmic domain	-	Wild-type Tar	0 mM NaCl	3	1000
		Wild-type Tar	50 mM NaCl	3	1000

Supplementary Table 8. Structural restraints applied in modelling of Tar structure

Stage	Duration (ns)	Restraints (kcal/mol nm^2)		
		Terminal residues of periplasmic domain[a]	TM helix[b]	TM cross-linking[c]
Simulation in implicit solvent	30	0<br>│<br>2000	0<br>│<br>2000	0<br>│<br>2000
Equilibration of explicit lipid and water	60	-	-	-
Simulation in explicit solvent	60	2000<br>│<br>250	2000<br>│<br>0	2000<br>│<br>0

[a]The terminal residues of Tar periplasmic domain were used as the distance restraints to mimic the piston motion upon ligand binding.

[b]Distance restraints for α-helix (N-O hydrogen bonds) at TM helices. The flat-bottomed region for α-helix restraints is between 2.8 to 3.5 Å and the potential is harmonic outside.

[c]Restraints derived from the previous disulfide cross-linking experiments (Supplementary Ref. 12). The flat-bottomed region for the Cβ-Cβ distance restraints is between 4.0 and 5.0 Å and the potential is harmonic outside.

Supplementary Table 9. Structure restraints applied before final MD simulations for mutant models

Stage	Duration (ns)	Restraints (kcal/mol nm^2)	
		Protein heavy atom position restraints	Protein backbone atom position restraints
Equilibration of explicit lipid and water	40	1000	-
Equilibration of explicit lipid and protein side chain	40	-	1000 → 0

Supplementary Table 10. Structural restraints applied during final MD simulation for the wild-type and mutant Tar

Stage	Duration (ns)	Restraints (kcal/mol nm^2)			
		Terminal residues of periplasmic domain	TM helix	TM cross-linking	C-termini of MH bundle[a]
MD production run in explicit solvent	800	250	0	0	250

[a]Restraints on the C-termini of MH bundle were only applied on the models containing the MH bundle.
The definitions for restraints of periplasmic domain, TM helix and TM cross-linking are the same as Supplementary Table 8.

Supplementary Methods

Details of MD simulations

Structure construction and MD simulations for the wild-type Tar and receptors with TM2 mutations

Following previous work by Park et al[1], simulations were performed for a structure that includes the periplasmic domain and transmembrane helices of *E. coli* Tar and the HAMP domain of receptor Af1503[9] from *Archaeoglobus fulgidus*. The hybrid receptor that carries the Af1503 HAMP domain instead of the native HAMP domain of Tar was already experimentally shown to transmit chemotactic signals[9]. Moreover, there is no experimentally determined structure of the native HAMP domain of Tar and the quality of the homology modelling for the Tar HAMP domain is not sufficiently high for MD simulations, due to the low sequence identity (about 25%) between the structures of *E. coli* and *A. fulgidus*.

The structures of the *E. coli* wild-type Tar periplasmic domain in the AA and AH states were constructed as described previously[1]. Briefly, the crystal structures are available for the AA (PDB ID: 1VLS)[10] and HH (both binding pockets are bound with Asp, holo-holo, PDB ID: 1VLT)[10] states of the periplasmic domain of Tar from *Salmonella* Typhimurium. *Salmonella* Tar AH model was constructed by superimposing one monomer of AA periplasmic domain onto HH periplasmic domain. Based on these models for *Salmonella* Tar, highly reliable homology models for *E.*

coli Tar (67% sequence identity with *Salmonella* Tar) AA and AH states were constructed using MODELLER 9.14[11]. Only a small part of *E. coli* Tar periplasmic domain (residues 56-165 were omitted) was included in the simulation system and restrained spatially. The transmembrane helices in the initial structure of *E. coli* Tar dimer were modeled based on the previous disulfide cross-linking results[1,12].

During the model building, all-atom models for the periplasmic domain and transmembrane helices of AA and AH states of wild-type Tar were firstly constructed using implicit solvent MD simulations with GROMACS 4.5.5 package[13]. Spatial restraints in the periplasmic domain, distance restraints for α-helix (N-O hydrogen bonds), and restraints based on TM cross-linking results were increased gradually from 0 to 2000 kcal/mol nm^2 (Supplementary Table 8). Afterward, the HAMP domain of Af1503 (PDB ID: 2L7H)[9] was attached to the modeled transmembrane helices of Tar. The transmembrane protein models were embedded into a pre-constructed explicit lipid bilayer with 128 DPPC[14] using LAMBADA and InflateGRO2[15]. The system was equilibrated using explicit solvent MD simulations. MD simulations were performed using GROMACS 4.5.5 package[13]. The system was described using SPC water[16] and united atom lipid model[17] with GROMOS96 forcefield[18]. The AA and AH systems were prepared separately, while the lower limit for the distance between protein and its periodic images was set to 25 Å. Both systems were slowly allowed to warm up under NPT conditions to 323 K using Berendsen thermostat[19] with the coupling constant of 0.1 ps and the semiisotropical Berendsen barostat[19] at 1 bar with the coupling constant of 1 ps and compressibility of 4.5×10^{-5} bar^{-1} in 200 ps, and

further equilibrated for 60 ns with 2 fs time step for lipids relaxation while protein was position-restrained. Under the same thermostat and barostat, the systems were further equilibrated for 60 ns with gradually decreased spatial restraints on the periplasmic domain (from 2000 to 250 kcal/mol nm^2), helix restraints (from 2000 to 0 kcal/mol nm^2) , and cross-linking distance restraints (from 2000 to 0 kcal/mol nm^2) (Supplementary Table 8, Supplementary Fig. 5a). There are no restraints on C-termini of HAMP domain.

For final simulations, three independent MD simulations were conducted for the AA and AH states of wild-type Tar for 800 ns with different initial velocity. The systems were coupled to Nose-Hoover thermostat[20] at 323 K with the coupling constant of 0.1 ps and the semiisotropical Parrinello-Rahman barostat[21] at 1 bar with 1 ps and compressibility of 4.5×10^{-5} bar^{-1}. To enable 2 fs time steps, bonds involving hydrogen atoms were constrained to equilibration length using the LINCS algorithm[22]. A real-space cutoff of 12 Å was used for the electrostatic and Lennard-Jones forces. The restraints used during the production of simulations are shown in Supplementary Table 10.

Homology modeling (MODELLER 9.14)[11] was performed to generate the AA and AH models of Tar^{TM2-1}, Tar^{TM2+1I}, and Tar^{TM2+2I} using the AA and AH models of wild-type Tar as the templates. While constructing the models using MODELLER, helix restraints in TM2 and TM2' and spatial restraints in periplasmic domain were applied. It should be noted that for the template, only Cα atoms in TM2 and TM2' were used to assure the quality of α-helix. The models for a specific mutant

generated by Modeller are very similar with Cα RMSD less than 1 Å. For each AA and AH state of Tar^{TM2-1}, Tar^{TM2+1I} and Tar^{TM2+2I}, three best models generated from homology modelling were selected based on Discrete Optimized Protein Energy (DOPE) values and used for MD simulations. Each structure of AA and AH state of Tar^{TM2-1}, Tar^{TM2+1I} and Tar^{TM2+2I} was equilibrated with protein position restrained (force constant 1000 kcal/mol nm^2) for 40 ns and then backbone restrained for 40 ns (force constant decrease gradually from 1000 kcal/mol nm^2 to 0) in explicit solvent lipid bilayers (Supplementary Table 9). Finally, production run for each model of Tar^{TM2-1}, Tar^{TM2+1I} and Tar^{TM2+2I} was performed (Supplementary Table 7). The restraints used during these simulations are shown in Supplementary Table 10. For the mutant receptors, positions of the terminal residues of periplasmic domain were also used as the restraints, to mimic the piston motion upon ligand binding. Here we assumed that because the TM2 mutation is below the periplasmic domain, ligand binding should elicit similar conformational changes in the periplasmic domain of the mutant receptor. There are no helix restraints applied during the final simulations.

To ensure that our results are qualitatively independent on the type of lipid, temperature, or on the length of the simulated receptor fragment, MD simulations were also performed for the wild-type Tar and Tar^{TM2+2I} fragments that contain additional *E. coli* Tar methylation helix (MH) bundle connecting the HAMP domain with another widely used lipid phosphatidylcholine (POPC)[23,24] at 300 K. The details for this part of MD simulations are described below.

Structure construction and MD simulations for the wild-type Tar and receptors with mutations after the HAMP domain

a) MD simulations in DPPC at 323 K

The simulation system contains a part of the periplasmic domain and transmembrane helices of *E. coli* Tar, Af1503 HAMP domain, and *E. coli* Tar methylation helix bundle. The model of *E. coli* Tar MH bundle was generated by homology modeling from the structure of Tsr methylation helix bundle (PDB ID:3ZX6)[25]. For mutants Tar^{HAMP_GVQPM}, Tar^{HAMP_TLPRY}, and Tar^{HAMP_VVPAY}, the residues in the linker region were mutated using UCSF Chimera[26]. The position restraints (250 kcal/mol nm^2 in *x-y* plane parallel to membrane) were applied to the cytoplasmic tip of this structure (Cα atoms of residues 328-332, 328'-332', 443-447 and 443'-447') during simulations (Supplementary Table 10). These atoms are still free to move in *z* direction thus making inward or outward movement possible. As the nearest distance between the region of interest (residues 267-297 of MH1 and MH1' and residues 485-509 of MH2 and MH2', where we analyzed for RMSF) and the restrained atoms of C-termini is about 80 Å, this restrain has little influence on the dynamics of the region of interest. Moreover, previously study showed that for the hybrid receptor of *E. coli* Tar and *Pseudomonas aerurginosa* Aer2, the difference in the mean of distance distribution for the residues E270, S298 and S487 in the Tar MH bundle between the kinase-on and kinase-off state is less than 10 Å[27], which is much smaller than 80Å. Three independent MD simulations of different initial structures generated from homology modelling were performed for the AA and AH models of

each receptor (Supplementary Table 7). The same MD parameters were used as for TM2 mutants.

b) MD simulations in POPC at 300 K

We constructed and conducted MD simulations for the AA and AH states of wild-type Tar, Tar^{TM2+2I} and Tar^{HAMP_GVQPM} with a part of the periplasmic domain and transmembrane helices of *E. coli* Tar, Af1503 HAMP domain, and *E. coli* Tar MH bundle in POPC at 300 K. Three independent MD simulations of different initial structures generated from homology modelling were performed for each model (Supplementary Table 7). All the other parameters are the same as specified above.

MD simulations of the periplasmic domain of Tar in presence of NaCl

MD simulations were performed for the dimer of *E. coli* Tar periplasmic domain (residues 36-180) using GROMACS 4.5.5 package[13]. The initial structure was prepared based on the structure of *Salmonella* Tar periplasmic domain dimer (PDB ID: 1VLS)[10] as the template. MODELLER 9.14[11] was used for homology modeling. The TIP3P[28] water model and AMBER99SB forcefield[29] was used to describe the system. The system was immersed in a dodecahedral water box while the lower limit for the distance between the atoms of the peptide and the edge of the box was set to 15 Å. By replacing solvent molecules, Na^+ or Cl^- ions were added to maintain the electrostatic neutrality and further NaCl was added to represent specific ionic strength. The system

was minimized using the steepest descent minimization approach. The system was then successively equilibrated in the NVT (300 K) and NPT (300 K and 1 atm) ensemble with all heavy atom restrained with a force constant of 1000 kcal/mol nm^2. Both equilibrations were performed for 5 ns with a time step of 1 fs. For the production run, the system was maintained at 300 K using V-rescale thermostat with a coupling constant of 0.1 ps and 1 atmosphere using a Parrinello-Rahamn barostat[21] with the coupling constant set to 2.0 ps. To enable 2 fs time steps, bonds involving hydrogen atoms were constrained to equilibration length using the LINCS algorithm[22]. A real-space cutoff of 10 Å was used for the electrostatic and Lennard-Jones forces.

Analyses of MD simulations

All the simulations were analyzed using GROMACS utilities[13] with custom written scripts. The structures were prepared using PyMol[30]. The time-evolution of secondary structure for the junction residues ^{211}GIRRMLLT^{218} in the wild-type and mutant Tar receptors was analyzed using the DSSP program[31]. The time evolution of helix curvature profile was calculated using the HELANAL module of MDAnalysis[32-34]. For the protocol of RMSF calculation, clustering of MD trajectories based on 3Å Cα RMSD was conducted first, and the middle structure of the largest cluster was used as the reference to calculate the RMSF of all the Cα of each residue.

Supplementary References

1 Park, H., Im, W. & Seok, C. Transmembrane signaling of chemotaxis receptor Tar: insights from molecular dynamics simulation studies. *Biophys. J.* **100**, 2955-2963 (2011).

2 Buron-Barral, M. C., Gosink, K. K. & Parkinson, J. S. Loss- and gain-of-function mutations in the F1-HAMP region of the *Escherichia coli aerotaxis* transducer Aer. *J. Bacteriol.* **188**, 3477-3486 (2006).

3 Sourjik, V. & Berg, H. C. Functional interactions between receptors in bacterial chemotaxis. *Nature* **428**, 437-441 (2004).

4 Bi, S., Pollard, A. M., Yang, Y., Jin, F. & Sourjik, V. Engineering hybrid chemotaxis receptors in bacteria. *ACS Synth. Biol.* **5**, 989-1001 (2016).

5 Gosink, K. K., Buron-Barral, M. C. & Parkinson, J. S. Signaling interactions between the aerotaxis transducer Aer and heterologous chemoreceptors in *Escherichia coli*. *J. Bacteriol.* **188**, 3487-3493 (2006).

6 Schulmeister, S., Grosse, K. & Sourjik, V. Effects of receptor modification and temperature on dynamics of sensory complexes in *Escherichia coli* chemotaxis. *BMC Microbiol.* **11**, 222 doi:10.1186/1471-2180-11-222 (2011).

7 Ames, P., Studdert, C. A., Reiser, R. H. & Parkinson, J. S. Collaborative signaling by mixed chemoreceptor teams in *Escherichia coli*. *Proc. Natl. Acad. Sci. USA* **99**, 7060-7065 (2002).

8 Paulick, A. *et al.* Mechanism of bidirectional thermotaxis in *Escherichia coli*. *Elife* **6,** e26607, doi: https://doi.org/10.7554/eLife.26607 (2017).

9 Hulko, M. *et al.* The HAMP domain structure implies helix rotation in transmembrane signaling. *Cell* **126**, 929-940 (2006).

10 Yeh, J. I. *et al.* High-resolution structures of the ligand binding domain of the wild-type bacterial aspartate receptor. *J. Mol. Biol.* **262**, 186-201 (1996).

11 Sali, A. & Blundell, T. L. Comparative protein modelling by satisfaction of spatial restraints. *J. Mol. Biol.* **234**, 779-815 (1993).

12 Pakula, A. A. & Simon, M. I. Determination of transmembrane protein structure by disulfide cross-linking: the *Escherichia coli* Tar receptor. *Proc. Natl. Acad. Sci. USA* **89**, 4144-4148 (1992).

13 Hess, B., Kutzner, C., van der Spoel, D. & Lindahl, E. GROMACS 4: algorithms for highly efficient, load-balanced, and scalable molecular simulation. *J. Chem. Theory Comput.* **4**, 435-447 (2008).

14 Berger, O., Edholm, O. & Jähnig, F. Molecular dynamics simulations of a fluid bilayer of dipalmitoylphosphatidylcholine at full hydration, constant pressure, and constant temperature. *Biophys. J.* **72**, 2002-2013 (1997).

15 Schmidt, T. H. & Kandt, C. LAMBADA and InflateGRO2: efficient membrane alignment and insertion of membrane proteins for molecular dynamics simulations. *J. Chem. Inf. Model.* **52**, 2657-2669 (2012).

16 Berendsen, H. J. C., Postma, J. P. M., van Gunsteren, W. F. & Hermans, J. Interaction models for water in relation to protein hydration. *In Intermolecular Forces*. Dordrecht, Holland. 331–342 (1981)

17 Tieleman, D. P., Sansom, M. S. & Berendsen, H. J. Alamethicin helices in a bilayer and in

solution: molecular dynamics simulations. *Biophys. J.* **76**, 40-49 (1999).

18 van Gunsteren, W. F. *et al.* Biomolecular simulation: the GROMOS96 manual and user guide. ETH Zürich, Zürich (1996).

19 Berendsen, H. J., Postma, J. v., van Gunsteren, W. F., DiNola, A. & Haak, J. Molecular dynamics with coupling to an external bath. *J. Chem. Phys.* **81**, 3684-3690 (1984).

20 Nosé, S. A unified formulation of the constant temperature molecular dynamics methods. *J. Chem. Phys.* **81**, 511-519 (1984).

21 Parrinello, M. & Rahman, A. Polymorphic transitions in single crystals: A new molecular dynamics method. *J. Appl. phys.* **52**, 7182-7190 (1981).

22 Hess, B., Bekker, H., Berendsen, H. J. & Fraaije, J. G. LINCS: a linear constraint solver for molecular simulations. *J. Comput. Chem.* **18**, 1463-1472 (1997).

23 Orekhov, P. S. *et al.* Signaling and adaptation modulate the dynamics of the photosensoric complex of *Natronomonas pharaonis*. *PLoS Comput. Biol.* **11**, e1004561 doi: 10.1371/journal.pcbi.1004561 (2015).

24 Ulmschneider, M. B. *et al.* Molecular dynamics of ion transport through the open conformation of a bacterial voltage-gated sodium channel. *Proc. Natl. Acad. Sci. USA* **110**, 6364-6369 (2013).

25 Ferris, H. U., Zeth, K., Hulko, M., Dunin-Horkawicz, S. & Lupas, A. N. Axial helix rotation as a mechanism for signal regulation inferred from the crystallographic analysis of the *E. coli* serine chemoreceptor. *J. Struct. Biol.* **186**, 349-356 (2014).

26 Pettersen, E. F. *et al.* UCSF Chimera—A visualization system for exploratory research and analysis. *J. Comput. Chem.* **25**, 1605-1612 (2004).

27 Samanta, D., Borbat, P. P., Dzikovski, B., Freed, J. H. & Crane, B. R. Bacterial chemoreceptor dynamics correlate with activity state and are coupled over long distances. *Proc. Natl. Acad. Sci. USA* **112**, 2455-2460 (2015).

28 Jorgensen, W. L., Chandrasekhar, J., Madura, J. D., Impey, R. W. & Klein, M. L. Comparison of simple potential functions for simulating liquid water. *J. Chem. Phys.* **79**, 926-935 (1983).

29 D.A. Case, *et al.* AMBER 2016. *University of California, San Francisco.* (2016).

30 Schrodinger, L. The PyMOL molecular graphics system, version 1.8. (2015).

31 Kabsch, W. & Sander, C. Dictionary of protein secondary structure: pattern recognition of hydrogen-bonded and geometrical features. *Biopolymers* **22**, 2577-2637 (1983).

32 Michaud-Agrawal, N., Denning, E. J., Woolf, T. B. & Beckstein, O. MDAnalysis: a toolkit for the analysis of molecular dynamics simulations. *J. Comput. Chem.* **32**, 2319-2327 (2011).

33 R. J. Gowers, *et al.* MDAnalysis: A Python package for the rapid analysis of molecular dynamics simulations. In S. Benthall and S. Rostrup editors, Proceedings of the 15th Python in Science Conference, pages 102-109, Austin, TX, 2016. SciPy.

34 Bansal, M., Kumar, S. & Velavan, R. HELANAL: a program to characterize helix geometry in proteins. *J. Biomol. Struct. Dyn.* **17**, 811-819 (2000).
